# Beer and wine consumption and risk of knee or hip osteoarthritis: a case control study

**DOI:** 10.1186/s13075-015-0534-4

**Published:** 2015-02-05

**Authors:** Stella G Muthuri, Weiya Zhang, Rose A Maciewicz, Kenneth Muir, Michael Doherty

**Affiliations:** Division of Epidemiology and Public Health, University of Nottingham, Clinical Sciences Building, Nottingham City Hospital, Nottingham, NG5 1PB UK; Academic Rheumatology, University of Nottingham, Clinical Sciences Building, Nottingham City Hospital, Nottingham, NG5 1PB UK; Respiratory, Inflammation, Autoimmunity Innovative Medicines, AstraZeneca AB, SE-432 83 Mölndal, Sweden; Institute of Population Health, University of Manchester, Manchester, UK; Division of Health Sciences, Warwick Medical School, Warwick University, Coventry, CV4 7AL UK

## Abstract

**Introduction:**

The aim of this study was to investigate the association between alcoholic and non-alcoholic beverages and knee or hip osteoarthritis (OA).

**Methods:**

We conducted a case–control study of Caucasian men and women aged 45 to 86 years of age from Nottingham, UK. Cases had clinically severe symptoms and radiographic knee or hip OA; controls had no symptoms and no radiographic knee or hip OA. Exposure information was sought using interview-based questionnaires and a semi-quantitative food frequency questionnaire to assess beverage consumption at ages 21 to 50 years. Odds ratios (ORs), adjusted ORs (aORs), 95% confidence intervals (CI) and *P* values were estimated using logistic regression models.

**Results:**

A total of 1,001 knee OA, 993 hip OA and 933 control participants were included in the study. Increasing beer consumption was associated with an increasing risk of OA (*P* for trend ≤0.001). Compared to those who did not consume beer, aORs for people who consumed 20 or more servings of beer were 1.93 (95% CI 1.26 to 2.94) and 2.15 (95% CI 1.45 to 3.19) for knee OA and hip OA, respectively. In contrast, increasing levels of wine consumption were associated with decreased likelihood of knee OA (*P* for trend <0.001). Compared to those who did not consume wine, aOR for knee OA among those who consumed 4 to 6 glasses of wine per week and ≥7 glasses of wine per week was 0.55 (95% CI 0.34 to 0.87) and 0.48 (95% CI 0.29 to 0.80), respectively. No association was identified between non-alcoholic beverages and knee or hip OA.

**Conclusions:**

Beer consumption appears to be a risk factor for knee and hip OA whereas consumption of wine has a negative association with knee OA. The mechanism behind these findings is speculative but warrants further study.

**Electronic supplementary material:**

The online version of this article (doi:10.1186/s13075-015-0534-4) contains supplementary material, which is available to authorized users.

## Introduction

Osteoarthritis (OA) is a common complex disorder with a number of recognised genetic, constitutional and environmental risk factors [1]. A major, potentially modifiable environmental lifestyle factor is nutrition. However, research with respect to dietary risk for OA has focused mainly on overall nutritional intake that influences body mass index (BMI), an increase in which is an accepted and important risk factor for knee OA and to a lesser extent hip and hand OA [2-4]. Although an increase in biomechanical stress on joint tissues is an obvious explanation for overweight and obesity as a risk factor for OA [5], it is possible that increased adiposity may also be deleterious for cartilage and other joint tissues via systemic metabolic changes, for example, altered adipokine secretion [6].

However, although many patients are interested in individual nutrients as possible risk factors for their OA, there are few studies that have examined this. There are data to suggest that vitamins and antioxidants are beneficial, especially for reducing structural progression of OA [7,8]. Several studies have included alcohol consumption as one of the potential risk factors for OA [9-12], of which only one found that alcohol consumption associated with an increased risk of development of OA [9]. Whether alcohol is a risk factor for OA, therefore, remains unclear and whether certain types of alcoholic drinks may relate to OA more than others has not been investigated. This case control study was undertaken in the Genetics of OA and Lifestyle (GOAL) database in which dietary and beverage data were available as part of the assessment of people with clinically severe knee or hip OA and of controls with no knee or hip OA.

## Methods

### Study population

The GOAL study was a case–control study of 3,171 participants (1,042 index knee OA cases, 1,006 index hip OA cases and 1,123 non-OA controls). The study was approved by the Nottingham Research Ethics Committee and fully informed consent was obtained from each participant.

The recruitment of cases and controls took place between 2002 and 2006 and details of the study are published elsewhere [13,14]. In brief, participants were Caucasian men and women, between 45- and 86-years old and resident in Nottinghamshire. Cases were selected if they had clinically significant symptomatic OA of the hip or knee, sufficient to warrant hospital referral. They were recruited if they had either undergone joint replacement, were on the orthopaedic waiting list or had been referred with symptomatic knee OA to the Nottingham Knee OA clinic. Controls were recruited from hospital intravenous urography (IVU) waiting lists and frequency matched to cases by age (±2 years) and gender. Controls with no OA symptoms and no evidence of hip OA on screening their IVU radiographs and no evidence of knee OA on fresh knee radiographs were invited to take part in the study.

### Case definition

New knee, hand and pelvis radiographs were taken at baseline assessment unless the participant had undergone radiography within two years of entry to the study or had undergone total joint replacement (TJR). Radiographic assessment and grading for features of OA have been described in detail elsewhere [13,14]. For this study we included symptomatic OA cases with Kellgren and Lawrence (KL) grades of ≥3 at the knee and ≥2 at the hip and asymptomatic controls with no radiographic evidence of hip or knee OA (defined as KL ≤2 at the knee and KL ≤1 at the hip).

### Exposure assessment

An interview-administered questionnaire and clinical examination were used to collect data. We assessed weekly alcohol consumption with a separate questionnaire with questions about beer, wine and spirits. This questionnaire was administered during an interview. Participants who reported drinking beer, wine and/or spirits on a regular basis (that is, at least one alcoholic drink per week) were asked to report the number of years and the average number of alcoholic drinks they consumed each week from the age of 10 to 15 years onwards. We measured units of alcohol by half pints for beer/lager/cider; a glass for white/red wine or sherry/martini; and tots for spirits, for example, gin, brandy, whisky, vodka (tots of spirits consumed at home were counted as a double). We then used information on the duration of alcohol consumption and the type of alcoholic beverage (that is, beer, wine and/or spirits) consumed by each individual across three decades: at ages 21 to 30, 31 to 40 and 41 to 50, to determine alcohol intake during adult life. For each type of beverage, a weighted number of drinks consumed per week was estimated, with weights equal to the number of years of consumption at each age period. For example, if a participant reported drinking an average of ten half-pints of beer per week for ten years at ages 21 to 30 and six half-pints of beer per week for eight years at ages 31 to 40, their weighted number of beer consumed per week at these ages were 10 and 4.8, respectively. We then summed up the weighted number of each type of beverage consumed at different ages to calculate the mean total alcohol intake during adult life. If an individual did not consume at least one alcoholic drink per week during a certain age period or had missing data on alcohol consumption, the amount of alcohol consumed for the available age periods was averaged. Beverage-specific estimates for beer, wine and spirits were also computed using the same procedures.

We sought information on non-alcoholic beverages (tea, coffee and fruit juice) using a self-administered 126 food item semi–quantitative food frequency questionnaire (FFQ). This FFQ was modified from the European Prospective Investigation of Cancer and Nutrition (EPIC) FFQ [15] and was used to assess habitual dietary intake during the fourth decade, that is, at ages 30 to 40 years. To elicit information for each food item, participants were asked to indicate their average frequency of consumption during their 30s in terms of a medium serving, a common household unit (for example, teaspoon, bowl, cup, glass) or portion size of each food or beverage (for example slice of bread, one egg) by checking one of the nine frequency categories which ranged from ‘never or less than once per month’ to ‘more than six times per day’.

The participants were re-contacted in January 2008 and further asked to complete a postal questionnaire to assess the reliability and validity of the data previously obtained. Of 3,022 participants from the GOAL study who were known to be still alive and living in the UK, 2,172 responded to the second questionnaire. As part of the expanded second questionnaire, participants also randomly received one of the seven food group tables (that is, meat and fish; bread and potatoes; fruits and vegetables; dairy products; drinks; sweets and snacks; or soup and lifetime patterns of food consumption) from the baseline FFQ. The drinks table was slightly modified to include four alcoholic beverages (beer, wine, sherry/martini and spirits) as these were not part of the 126 food item semi-quantitative FFQ. Servings for units of alcoholic beverages were as follows: half pints for beer/lager/cider; a glass for white/red wine; a glass of sherry/vermouth/liqueurs; and tots for spirits, for example, gin, brandy, whisky, vodka. In total 2,117 of 3,022 participants completed food frequency tables and data were used to assess the agreement on alcoholic and non-alcoholic intake during the fourth decade of life.

### Confounding factors

The questionnaire collected information on socio-demographic factors, employment history, occupational activity, history of physical activity, significant injury, medical conditions and also contained detailed questions on other risk factors for OA. Weight (kg) and height (cm) were measured by a trained research nurse during the clinical examination.

For this analysis, we considered age (years), gender (male versus female), BMI (kg/m^2^), social class, smoking (never, past, current smoker), history of joint injury (yes or no), self-report of gout (yes or no), and kidney disease (yes or no) and energetic leisure-time physical activity (yes or no). Social class was classified as I/II, III, IV and V/VI using the Standard Occupational Classification based on the longest job [16,17]. Occupational exposure was scored as 0 to 5, with 1 point each for: kneeling, squatting, performing heavy work while standing for one hour or more per week, lifting 25 kg ten or more times per week, and lifting 50 to 100 kg at least once per week. Participants were divided into those without any risks and those with a score of one or more. Leisure-time energy physical activity was defined as ever participating in activities that increased heart rate and produced sweating, such as swimming, jogging, running and so on, for 20 minutes or more at least three times per week.

### Statistical analyses

We first assessed the agreement on alcoholic and non-alcoholic intake during the fourth decade of life on the basis of interview-administered questionnaire, original FFQ and recalled FFQ tables. Weighted kappa (quadratic) statistics between original and recalled FFQ tables for alcoholic drinks ranged from 0.31 to 0.80 (spirits = 0.31, wine = 0.47, beer = 0.80) while for non-alcoholic drinks ranged from 0.37 to 0.56 (pure fruit juice = 0.37, coffee = 0.55, tea = 0.56).

We used unconditional logistic regression models to estimate odds ratio (OR) and 95% confidence intervals (CI). These were examined separately for knee and hip OA. Firstly, we estimated the average number of units of alcohol consumed per week and subsequently categorised weekly units of alcohol into five groups: non-drinkers, ≤2 drinks per week, >3 to 6 per week, >7 to 14 drinks per week and ≥15 drinks per week. Secondly, we categorised each beverage consumed into five frequency groups based on the distribution among controls and used the lowest category as the referent category in the analysis. We used two multivariate models to adjust for potential confounders. The first model adjusted for the following factors selected *a priori*: age (years), gender (men versus women), BMI (<25 kg/m^2^, 25 to 30 kg/m^2^, >30 kg/m^2^) and smoking (never, past or current smoker). The second model adjusted for additional variables including occupational exposure (no risk factors, 1, 2 and 3 or more knee occupational risk factors), energetic leisure time physical activities at ages 20, 30 or 40 (yes or no), history of significant joint injury (yes or no), kidney disease (yes or no), gout (yes or no) and other beverages, as appropriate. We also assessed trends in OA risk for a given beverage by fitting it as a categorical variable.

We also examined potential effect modification by gender (men versus women) and cigarette smoking status (ever smoker yes versus no) by comparing the log-likelihood statistic for models that used the cross-product interaction term in the logistic regression models to those without and then assessed the statistical significance of the interaction using a likelihood ratio test [18].

In addition, we repeated analysis to examine average alcohol consumption at different age periods: 21 to 30, 31 to 40 and 41 to 50 and association with OA. For this analysis each period was examined separately to evaluate whether timing of alcohol exposure was associated with OA risk.

All analyses were performed using STATA version 13 software. *P*-values (two-tailed) are reported.

## Results

### Study subjects

In total 1,994 symptomatic OA cases (1001 knee OA and 993 hip OA) with radiographic evidence and 933 asymptomatic controls with no radiographic evidence of knee or hip OA as defined were included in this study. Table 1 shows the characteristics of the study population. Overall, cases were significantly older than controls. They were also more likely to be obese and from lower social economic groups, to report previous joint injury, occupational risk factors, gout and cardiovascular diseases, such as hypertension and heart disease and diuretic use. However, they were less likely than controls to be current smokers or to report kidney problems.

**Table 1:** **Demographic characteristics of study participants**

**Risk factor**	**Control, number = 933**		**Knee OA, number = 1001**		**Hip OA, number = 993**		
	**Number**	**%**	**Number**	**%**	**Number**	**%**	***P*** **value** ^**a**^
**Age, mean (SD)**	63.4	(8.5)	68.3	(7.3)	67.6	(7.1)	<0.001
**Gender, Male**	484	51.9	519	51.9	494	49.8	0.556
**Body mass Index (BMI)**							
BMI, mean (SD)	27.3	(4.5)	31.2	(5.3)	29.3	(5.2)	<0.001
<25 kg/m^2^	308	33.0	90	9.0	191	19.2	<0.001
>25 to <30 kg/m^2^	396	42.4	363	36.3	413	41.6	
≥30 kg/m^2^	229	24.5	547	54.7	389	39.2	
**Social Class**							<0.001
I and II	275	29.8	176	17.9	217	22.1	
III	211	22.8	204	20.7	235	23.9	
IV	261	28.3	361	36.6	302	30.7	
V and VI	177	19.2	245	24.9	230	23.4	
**Smoking**							<0.001
Never	317	34.0	441	44.1	391	39.4	
Past smoker	407	43.6	463	46.3	499	50.3	
Current smoker	209	22.4	97	9.7	103	10.4	
**Occupational risk factors**							0.522
No knee OA risk factors	471	50.5	529	53.0	537	54.2	
1 knee OA risk factor	145	15.7	140	14.0	146	14.7	
2 knee OA risk factors	142	15.2	160	16.0	155	15.6	
3 + knee OA risk factors	174	18.7	169	16.9	153	15.4	
**Significant joint injury**	157	16.8	332	33.2	221	22.3	<0.001
**Co-morbidities**							
Hypertension	339	36.3	519	51.9	451	45.5	<0.001
Heart disease	154	16.5	234	23.5	179	18.1	<0.001
Type 1 diabetes	11	1.2	22	2.2	7	0.7	0.013
Type 2 diabetes	63	6.8	92	9.2	69	7.0	0.077
Stroke/haemorrhage	45	4.8	65	6.5	57	5.8	0.281
Kidney disease	368	39.5	61	6.1	61	6.1	<0.001
Liver disease	34	3.6	22	2.2	34	3.4	0.137
Gout	62	6.6	109	10.9	93	9.4	0.004
**Diuretic use**	190	20.4	359	35.9	352	35.5	<0.001

^a^Χ^2^ test for categorical data; one-way analysis-of-variance (ANOVA) for continuous data. The number per group, mean (s.d.) or percentage prevalence are presented. OA, osteoarthritis; SD, standard deviation.

Overall, controls were more likely to report drinking alcohol on a regular basis (at least one alcoholic drink per week) during adult life (that is, ages 21 to 50 years) compared to OA cases. Among alcohol drinkers, a higher proportion of OA cases reported that they drank beer exclusively while a higher portion of controls drank a mixture of beer, wine and/or spirits (Table 2). As for non-alcoholic beverages, 86% of the population reported drinking tea at least once a day during the fourth decade of life whereas the proportion of those who drank coffee and pure fruit juice at least once a day during the same period was 58% and 13%, respectively.

**Table 2:** **Characteristics of the study participants according to beverage consumption during adult life (ages 21 to 50 years)**

**Exposure**	**Control, number = 933**	**Knee OA, number = 1001**	**Hip OA, number = 993**
**Alcohol consumption (ages 21 to 50)**			
Nondrinkers^a^, n (%)	186 (19.9)	280 (28.0)	257 (29.9)
Ever drinkers^b^, n (%)	747 (80.1)	721 (72.0)	736 (74.1)
**By type of alcohol**			
Drinkers of beer exclusively, n (%)	272 (29.1)	345 (34.5)	336 (33.8)
Drinkers of wine exclusively, n (%)	73 (7.8)	37 (3.7)	61 (6.1)
Drinkers of spirits exclusively, n (%)	33 (3.5)	38 (3.8)	29 (2.9)
Other drinkers^c^, n (%)	348 (37.3)	276 (27.6)	282 (28.4)
Missing data on alcohol type, n (%)	21 (2.3)	25 (2.5)	28 (2.8)
Alcoholic drinks per week, median (IQR)	10 (5 to 21)	11 (4 to 24)	10 (4 to 24)
**Non-alcohol consumption** ^**d**^ **(ages 30 to 40)**			
**Tea intake (cups)**			
never/3 times per month, n (%)	98 (10.5)	66 (6.6)	64 (6.5)
1 to 6 times per week, n (%)	48 (5.1)	51 (5.1)	38 (3.8)
≥1 per day, n (%)	775 (83.1)	876 (87.5)	877 (88.3)
**Coffee intake (cups)**			
never/3 times per month, n (%)	202 (21.7)	268 (26.8)	213 (21.5)
1 to 6 times per week, n (%)	152 (16.3)	174 (17.4)	174 (17.5)
≥1 per day, n (%)	567 (60.8)	551 (55.0)	592 (59.6)
**Pure fruit juice (glasses)**			
never/3 times per month, n (%)	511 (54.8)	586 (58.5)	579 (58.3)
1 to 6 times per week, n (%)	284 (30.4)	260 (26.0)	285 (28.7)
≥1 per day, n (%)	126 (13.5)	147 (14.7)	115 (11.6)

^a^Nondrinkers include never drinkers at ages 21 to 50 years. ^b^Ever drinkers drunk alcohol on a regular basis (at least 1 alcoholic drink per week) at ages 21 to 50 years. ^c^drinkers of a mixture of beer, wine, and / or spirits. ^d^Analyses are based on age period 30 to 40 years when information on non-alcoholic beverage consumption was assessed. The number per group and percentages, or median (interquartile range (IQR) are presented. n, number; OA, osteoarthritis.

### Association between alcohol consumption and OA

#### Knee OA

Table 3 summarises findings from unadjusted and adjusted analyses for the association between alcohol consumption during adult life (that is, ages 21 to 50 years) and the risk of knee OA. Results indicate that adjustment for potential confounding factors had some appreciable effect on the risk estimates. In the fully adjusted model, increased total alcohol intake per week was not statistically significantly associated with the risk of knee OA. The test for trend across categories of total alcohol intake was also non-significant. We then conducted separate analysis by gender to assess any residual confounding and found a dose–response relationship among men but no individual ORs were statistically significant. Moreover, test for heterogeneity of odds ratios was significant (*P* = 0.040) thereby suggesting that the association between alcohol consumption and risk of knee OA was different for men and women (Additional file 1: Table S1). We also observed a moderate association with alcohol intake of seven to fourteen units per week among ever smokers (aOR 0.56, 95% CI 0.34 to 0.92; *P* = 0.023) but not among never smokers (aOR 1.65, 95% CI 0.89 to 3.04; *P* = 0.110), *P* for interaction = 0.042 (data not shown).

**Table 3:** **Odds ratios for knee OA according to alcoholic and non-alcoholic beverage consumption**

**Beverage**	**Frequency of consumption**	**Knee OA**	**Controls**	**CRUDE ORs**				**Adjusted** ^**a**^ **OR**				**Adjusted** ^**b**^ **OR**			
		**Number**	**Number**	**ORs**	**95.0% CI**		***P*** **value**	**ORs**	**95.0% CI**		***P*** **value**	**ORs**	**95.0% CI**		***P*** **value**
**Alcoholic** ^**c**^															
Total alcohol intake															
	Nondrinkers^d^	280	186	1.00				1.00				1.00			
	< = 2 per week	102	93	0.73	0.52	1.02	0.065	0.90	0.61	1.31	0.571	0.81	0.53	1.24	0.340
	3 to 6 per week	135	168	**0.53**	**0.40**	**0.72**	**<0.001**	0.76	0.54	1.07	0.111	0.73	0.50	1.06	0.101
	7 to 14 per week	177	204	**0.58**	**0.44**	**0.76**	**<0.001**	0.95	0.68	1.32	0.745	0.82	0.57	1.20	0.309
	> = 15 per week	282	261	**0.72**	**0.56**	**0.92**	**0.009**	**1.43**	**1.01**	**2.02**	**0.042**	1.18	0.81	1.74	0.389
	*P* trend						**0.004**				0.082				0.514
Beer (half pints)	None^e^	388	340	1.00				1.00				1.00			
	< = 3 per week	128	130	0.86	0.65	1.15	0.309	1.09	0.78	1.51	0.617	1.02	0.70	1.47	0.929
	4 to 7 per week	94	139	**0.59**	**0.44**	**0.80**	**0.001**	0.92	0.64	1.33	0.674	0.90	0.60	1.37	0.630
	8 to 19 per week	183	162	0.99	0.77	1.28	0.938	1.73	**1.22**	**2.46**	**0.002**	**1.76**	**1.19**	**2.60**	**0.005**
	> = 20 per week	183	141	1.14	0.87	1.48	0.339	**2.32**	**1.59**	**3.38**	**<0.001**	**1.93**	**1.26**	**2.94**	**0.002**
	*P* trend						0.599				**<0.001**				**0.001**
Wine (glasses)	None^e^	770	579	1.00				1.00				1.00			
	< = 1 per week	39	43	0.68	0.44	1.07	0.093	0.69	0.42	1.13	0.139	0.72	0.40	1.28	0.259
	2 to 3 per week	77	110	**0.53**	**0.39**	**0.72**	**<0.001**	**0.68**	**0.48**	**0.97**	**0.031**	0.75	0.50	1.12	0.161
	4 to 6 per week	50	103	**0.37**	**0.26**	**0.52**	**<0.001**	**0.55**	**0.36**	**0.82**	**0.004**	**0.55**	**0.34**	**0.87**	**0.011**
	> = 7 per week	40	77	**0.39**	**0.26**	**0.58**	**<0.001**	**0.56**	**0.36**	**0.88**	**0.012**	**0.48**	**0.29**	**0.80**	**0.005**
	*P* trend						**<0.001**				**<0.001**				**<0.001**
Spirits (tots)	None^e^	756	654	1.00				1.00				1.00			
	< = 1 per week	48	43	0.97	0.63	1.48	0.872	1.03	0.64	1.67	0.895	1.16	0.66	2.02	0.602
	2 to 3 per week	60	76	**0.68**	**0.48**	**0.97**	**0.035**	0.76	0.51	1.14	0.189	0.79	0.50	1.26	0.330
	4 to 7 per week	58	64	0.78	0.54	1.14	0.198	0.99	0.65	1.50	0.954	0.77	0.48	1.23	0.272
	> = 8 per week	54	75	**0.62**	**0.43**	**0.9**	**0.011**	0.81	0.53	1.23	0.325	0.80	0.49	1.30	0.370
	*P* trend						**0.002**				0.262				0.151
**Non-alcoholic** ^**f,c**^															
Tea (cups)	= <1 per day	155	190	1.00				1.00				1.00			
	2 to 3 per day	261	271	1.18	0.90	1.55	0.231	1.04	0.76	1.43	0.784	0.97	0.67	1.41	0.892
	4 to 5 per day	366	290	1.55	**1.19**	**2.01**	**0.001**	1.32	0.98	1.79	0.071	1.34	0.93	1.92	0.114
	6+ per day	211	170	1.52	**1.14**	**2.04**	**0.005**	**1.46**	**1.04**	**2.05**	**0.027**	1.29	0.86	1.92	0.214
	*P* trend						**0.001**				**0.006**				0.050
Coffee (cups)	= <6 per week	442	354	1.00				1.00				1.00			
	1 per day	236	195	0.97	0.77	1.23	0.795	0.89	0.68	1.16	0.390	0.83	0.61	1.12	0.222
	2-3 per day	215	249	**0.69**	**0.55**	**0.87**	**0.002**	0.79	0.61	1.03	0.084	0.81	0.60	1.10	0.180
	= >4 per day	100	123	**0.65**	**0.48**	**0.88**	**0.005**	0.96	0.68	1.36	0.812	1.14	0.75	1.72	0.547
	*P* trend						**<0.001**				0.269				0.707
Pure fruit juice (glasses)	<1/month	454	368	1.00				1.00				1.00			
	1/month - 1/week	234	251	**0.76**	**0.60**	**0.95**	**0.015**	0.98	0.76	1.28	0.908	0.94	0.71	1.26	0.691
	2-6 per week	158	176	**0.73**	**0.56**	**0.94**	**0.015**	0.88	0.66	1.19	0.410	0.85	0.61	1.19	0.341
	> = 1 per day	147	126	0.95	0.72	1.24	0.690	1.02	0.74	1.40	0.919	0.99	0.69	1.42	0.958
	*P* trend						0.176				0.786				0.652

^a^adjusted for age, gender, BMI, smoking; ^b^adjusted for age, gender, BMI, smoking, other alcoholic/non-alcoholic beverages^c^, occupational risks, significant injury, kidney disease, energetic physical activity at ages 21 to 50 and gout. ^d^includes participants who did not drink alcohol on a regular basis (at least 1 alcoholic drink per week) during age period 21 to 50 years. ^e^includes alcohol abstainers at ages 21 to 50 and drinkers of other alcoholic drinks. ^f^Analyses are based on age period 30 to 40 years when information on non-alcoholic beverage consumption was assessed. CI, confidence interval; OA, osteoarthritis; OR, odds ratio.

When we evaluated whether the associations varied by type of alcohol, we found a positive significant association between beer intake and knee OA. Compared to those who never consumed beer, the adjusted ORs for knee OA among those who consumed 8 to 19 and 20 or more half-pints of beer per week was 1.76 (95% CI 1.19 to 2.60; *P* = 0.005) and 1.93 (95% CI 1.26 to 2.94; *P* = 0.002), respectively (Table 3). This effect increased with increasing levels of beer consumption (*P* for trend = 0.001). When stratified by gender, the association between heavier beer consumption (≥8 half-pints per week) and knee OA appeared to be limited to men, but there was no significant interaction (*P* = 0.588) between beer intake and gender (Additional file 1: Table S1).

More interestingly, there was an inverse dose response association between wine (that is, white/red wine or sherry/martini) consumption during adult life and knee OA, (Table 3). Compared to those who never drank wine, the adjusted ORs for those who drank 4 to 6 glasses and ≥7 glasses of wine per week was 0.55 (95% CI 0.34 to 0.87; *P* = 0.011) and 0.48 (95% CI 0.29 to 0.80; *P* = 0.005), respectively, with increasing levels of wine intake inversely associated with knee OA (*P* for trend <0.001). When stratified by gender, these significant associations remained in both men and women (Additional file 1: Table S1), but the interaction between wine intake and gender was not statistically significant (*P* = 0.331).

Conversely, intake of spirits (for example, gin, brandy, whisky, vodka) during adult life was not associated with the risk of knee OA (Table 3). Similarly there was no significant interaction between intake of spirits and gender (Additional file 1: Table S1). There were also no significant multiplicative interactions between each individual alcoholic beverage (beer, wine or spirits) and cigarette smoking (data not shown).

We evaluated whether the associations varied at different periods of alcohol consumption. Similar risk patterns, that is, positive association with beer but negative association with wine consumption, were observed at different periods of alcohol consumption although the significance level varied (Additional file 1: Table S3).

#### Hip OA

Table 4 displays results for the association between alcohol consumption during adult life (that is, ages 21 to 50 years) and the risk of hip OA. Compared to those who never consumed alcohol, adjusted ORs for hip OA among those who consumed two or fewer units of alcohol per day was 0.63 (95% CI 0.42 to 0.94; *P* = 0.025). However, there was no significant trend with increasing levels of alcohol consumption (*P* for trend = 0.265; Table 4). When stratified by gender, these associations were only seen among men (Additional file 1: Table S2). In addition, there were no statistically significant multiplicative interactions between alcohol consumption by gender or cigarette smoking (data not shown).

**Table 4:** **Odds ratios for hip OA according to alcoholic and non-alcoholic beverage consumption**

**Beverage**	**Frequency of consumption**	**Hip OA**	**Controls**	**CRUDE ORs**				**Adjusted** ^**a**^ **OR**				**Adjusted** ^**b**^ **OR**			
		**Number**	**Number**	**ORs**	**95.0% CI**		***P*** **value**	**ORs**	**95.0% CI**		***P*** **value**	**ORs**	**95.0% CI**		***P*** **value**
**Alcoholic** ^**c**^															
Total alcohol intake	Nondrinkers^d^	257	186	1.00				1.00				1.00			
	< = 2 per week	90	93	**0.70**	**0.50**	**0.99**	**0.044**	**0.66**	**0.46**	**0.96**	**0.031**	**0.63**	**0.42**	**0.94**	**0.025**
	3 to 6 per week	174	168	0.75	0.56	1.00	0.047	0.88	0.65	1.20	0.432	0.88	0.63	1.24	0.468
	7 to 14 per week	164	204	**0.58**	**0.44**	**0.77**	**<0.001**	0.83	0.60	1.13	0.232	0.75	0.53	1.06	0.106
	> = 15 per week	280	261	0.78	0.60	1.00	0.050	**1.42**	**1.03**	**1.96**	**0.030**	1.27	0.90	1.81	0.177
	*P* trend						**0.022**				0.062				0.265
Beer (half pints)	None^e^	384	340	1.00				1.00				1.00			
	< = 3 per week	119	130	0.81	0.61	1.08	0.153	0.84	0.62	1.15	0.285	0.79	0.56	1.11	0.169
	4 to 7 per week	121	139	0.77	0.58	1.02	0.072	1.13	0.82	1.55	0.460	1.15	0.81	1.64	0.435
	8 to 19 per week	156	162	0.85	0.65	1.11	0.236	**1.44**	**1.03**	**2.00**	**0.030**	**1.49**	**1.04**	**2.15**	**0.031**
	> = 20 per week	185	141	1.16	0.89	1.51	0.264	**2.36**	**1.65**	**3.37**	**<0.001**	**2.15**	**1.45**	**3.19**	**<0.001**
	*P* trend						0.703				**<0.001**				**<0.001**
Wine (glasses)	None^e^	703	579	1.00				1.00				1.00			
	< = 1 per week	37	43	0.71	0.45	1.11	0.136	0.68	0.42	1.09	0.111	0.67	0.39	1.14	0.140
	2 to 3 per week	102	110	0.76	0.57	1.02	0.069	0.83	0.60	1.14	0.242	0.97	0.68	1.38	0.868
	4 to 6 per week	61	103	**0.49**	**0.35**	**0.68**	**<0.001**	**0.63**	**0.44**	**0.91**	**0.013**	0.68	0.45	1.03	0.070
	> = 7 per week	62	77	**0.66**	**0.47**	**0.94**	**0.022**	0.91	0.62	1.33	0.624	0.88	0.58	1.34	0.549
	*P* trend						**<0.001**				0.052				0.181
Spirits (tots)	None^e^	756	654	1.00				1.00				1.00			
	< = 1 per week	43	43	0.87	0.56	1.34	0.514	0.86	0.54	1.38	0.534	1.01	0.60	1.71	0.972
	2 to 3 per week	73	76	0.83	0.59	1.16	0.283	0.91	0.63	1.31	0.612	0.90	0.60	1.35	0.610
	4 to per week	53	64	0.72	0.49	1.05	0.084	0.84	0.56	1.25	0.391	**0.63**	**0.41**	**0.98**	**0.040**
	> = 8 per week	40	75	**0.46**	**0.31**	**0.69**	**<0.001**	**0.59**	**0.39**	**0.91**	**0.017**	**0.53**	**0.33**	**0.85**	**0.008**
	*P* trend						**<0.001**				**0.020**				**0.002**
**Non-alcoholic** ^**f,c**^															
Tea (cups)	= <1 per day	150	190	1.00				1.00				1.00			
	2 to 3 per day	310	271	**1.45**	**1.11**	**1.90**	**0.007**	1.19	0.89	1.60	0.240	1.08	0.77	1.51	0.656
	4 to 5 per day	315	290	**1.38**	**1.05**	**1.80**	**0.019**	1.14	0.85	1.52	0.386	1.05	0.74	1.47	0.797
	6+ per day	204	170	**1.52**	**1.13**	**2.04**	**0.005**	1.36	0.99	1.88	0.058	1.18	0.81	1.71	0.389
	*P* trend						**0.019**				0.112				0.472
Coffee (cups)	= <6 per week	387	354	1.00				1.00				1.00			
	1 per day	269	195	1.26	1.00	1.59	0.051	1.12	0.88	1.44	0.358	1.11	0.85	1.47	0.438
	2 to 3 per day	243	249	0.89	0.71	1.12	0.329	0.95	0.74	1.21	0.675	0.97	0.74	1.28	0.850
	= >4 per day	80	123	**0.59**	**0.43**	**0.82**	**0.001**	0.79	0.56	1.12	0.183	0.86	0.58	1.30	0.483
	*P* trend						**0.004**				0.241				0.582
Pure fruit juice (glasses)	<1/month	429	368	1.00				1.00				1.00			
	1/month to 1 / week	265	251	0.91	0.73	1.13	0.381	1.06	0.84	1.35	0.607	1.01	0.78	1.31	0.918
	2 to 6 per week	170	176	0.83	0.64	1.07	0.145	1.05	0.80	1.38	0.743	1.14	0.84	1.54	0.413
	> = 1 per day	115	126	0.78	0.59	1.04	0.097	0.81	0.59	1.11	0.194	0.88	0.62	1.25	0.482
	*P* trend						**0.050**				0.397				0.880

^a^adjusted for age, gender, BMI, smoking; ^b^adjusted for age, gender, BMI, smoking, other alcoholic/non-alcoholic beverages^c^, occupational risks, significant injury, kidney disease, energetic physical activity at ages 21 to 50 and gout. ^d^includes participants who did not drink alcohol on a regular basis (at least one alcoholic drink per week) during age period 21 to 50 years. ^e^includes alcohol abstainers at ages 21 to 50 and drinkers of other alcoholic drinks. ^f^Analyses are based on age period 30 to 40 years when information on non-alcoholic beverage consumption was assessed. BMI, body mass index; CI, confidence interval; OA, osteoarthritis; OR, odds ratio.

Among individual alcoholic beverages, increasing beer consumption was associated with increased risk of hip OA. Compared to those who never consumed beer, the adjusted OR for those who drank 8 to 19 and 20 or more servings of beer per week was 1.49 (95% CI 1.04 to 2.15; *P* = 0.031) and 2.15 (95% CI 1.45 to 3.19; *P* <0.001), respectively, with ORs increased with increasing levels of beer consumption (*P* for trend <0.001). Test for heterogeneity of ORs was significant (*P* = 0.0298) suggesting that the effect of beer intake was significantly different for men and women. Stratification by gender revealed that the odds of hip OA was 1.8 times higher in men who consumed 20 or more half pints of beer per week (aOR 1.83 (95% CI 1.14 to 2.93); *P* = 0.012) whereas among women no significant associations were observed (Additional file 1: Table S2).

Interestingly, there was an inverse association between spirits and hip OA. The adjusted OR for those who consumed four to seven and eight or more units of spirits per week was 0.63 (95% CI 0.41 to 0.98) and 0.53 (95% CI 0.33 to 0.85), respectively. Correspondingly, there was a significant trend with increasing categories of spirits consumption (*P* for trend = 0.002). When stratified by gender, the inverse relation with spirits intake was significantly evident in men (Additional file 1: Table S2), but the interaction was not significant (*P* = 0.322). No statistically significant associations were observed between wine and risk of hip OA (Table 4). Moreover, test for interactions by cigarette smoking for each individual alcoholic beverage (beer, wine or spirits) were not statistically significant (data not shown).

When we examined different age periods separately, high beer consumption (20 or more half-pints of beer per week) at ages 21 to 30, 31 to 40 years and 41 to 50 years was associated with an increased risk of hip OA. In contrast, we found a decreased association with spirits consumption (eight or more units of spirits per week) at ages 31 to 40 years and 41 to 50 years (Additional file 1: Table S4).

### Associations between non-alcoholic beverages and OA risk

Information on non-alcoholic beverage intake was assessed when participants were in their 30s. For this analysis we included 2,893 participants (921 controls, 993 knee OA cases and 979 hip OA cases) who completed a semi-quantitative food frequency questionnaire.

Although increased tea intake seemed to be positively associated with knee OA, this association disappeared after adjusting for all potential confounding factors (Table 3). No clear association was observed with other non-alcoholic beverages and knee OA. Results were similar for hip OA (Table 4).

## Discussion

This is the first case–control study, involving approximately 3,000 participants, primarily designed to examine lifestyle risk factors for knee and hip OA including alcoholic and non-alcoholic beverages. Among alcoholic drinks, we found a dose–response, positive association between beer consumption and knee/hip OA. In contrast, consumption of wine was negatively associated with knee OA whereas spirits intake was negatively associated with hip OA. We also observed similar patterns of associations with alcohol consumption at specific time periods in adult life. No significant association with OA was identified for non-alcoholic beverages including tea, coffee or pure fruit juice for both knee and hip OA.

To the best of our knowledge, alcohol intake has not previously been investigated as a possible independent risk factor for either the development or progression of OA. Of the four studies [9-12] that we identified from the literature, all included alcohol as a potential confounding factor and the results are inconsistent. For example, a recent cohort study found no significant association between alcohol intake and hip OA after 22 years of follow up in 840 individuals in Finland [10]. Similarly, a cross sectional study of 568 women from the Nurses’ Health Study in the USA reported no significant association between self-reported hip replacement due to OA and alcohol intake [11]. However, one small hospital based one-year cohort study in 109 Japanese people with newly diagnosed knee OA found that alcohol associated with less functional disability [12]. In contrast, a population based cross sectional study in Greece found a positive association between alcohol intake and rheumatic complaints, predominantly back pain and OA, but unfortunately no separate analysis was presented for OA [9]. It is noteworthy that all these studies assessed alcohol consumption as a combined intake of beer, wine and spirits. They were not primarily designed for the question of alcohol consumption and risk of OA and, therefore, could not adequately address the question of interest. In contrast, GOAL was primarily designed for a case control analysis and powered for multiple risk factors including alcohol consumption. In addition to total alcohol intake, we undertook analyses for different type of alcohol and for other non-alcoholic drinks, such as coffee and tea. After simultaneously controlling for wine and spirits intake, energetic leisure activities, self-reported kidney disease and gout and other major established risk factors of OA, such as age, gender, BMI, occupational risk and joint injury, we found beer consumption to be a risk factor both for knee and hip OA with a demonstrated dose response effect. In contrast, wine was negatively associated with knee OA, suggesting that alcohol itself is not necessarily the factor that influences the risk of OA but that other factors contained within wine and beer may exert differential effects on the risk of OA.

As with some diseases including OA which have a long latency period, the effect of lifestyle exposures may occur many years before diagnosis. Moreover, the specific ages of susceptibility may also be important when estimating environmental and lifestyle exposures and the risk of disease. However, the proximal age of OA onset and age at dietary exposure in OA are still unknown. The complex nature of OA further presents a challenge for epidemiological studies because radiographic OA often occurs without symptoms. Nevertheless, considerable evidence has shown that the incidence of OA increases with age, with a higher preponderance in women after the age of 55 [19]. Other lines of evidence have illustrated that cumulative exposures at younger ages may be critical. For example, a study by Gelber *et al*. [20] found greater BMI at ages 20 to 29 in young men to be associated with the incidence of knee OA at age 65. Therefore, due to the retrospective nature of our study design, we considered average alcohol consumption during adult life (ages 21 to 50 years). Since alcohol intake may change during adulthood, we also further evaluated alcohol intake at three different time periods, namely, ages 21 to 30, 31 to 40 and 41 to 50. We first adjusted for risks factors selected *a priori* (age, sex, BMI and cigarette smoking) and further adjusted for other potentially important confounding factors (Tables 3 and 4; full models for the primary analysis are presented in Additional file 1: Tables S5 – S8). The results demonstrated similar patterns of the associations but varied at significant levels for both knee and hip OA.

We also performed *post-hoc* stratified analysis and found evidence of moderate significant interactions between alcohol consumption and knee OA by either gender or smoking. For example, a significant dose–response relationship was observed in men but not in women (Additional file 1: Table S1). It is possible that since a high proportion of non-alcohol drinkers were women (39% versus 12%), this may have diminished the power to detect a significant dose–response relationship in women. Our data also demonstrated an interaction between alcohol consumption and smoking in the development of knee OA, with the effect of moderate alcohol intake (7 to 14 units per week) limited to ever smokers. Recent meta-analyses [21,22] found a negative association between smoking and OA in studies where the control population were recruited from hospital settings. Therefore, the negative association of smoking suggested by our findings is likely to be explained by the selection of hospital controls who often have a higher exposure to smoking than the general population. Moreover, we assessed smoking status at the study baseline and did not take into account the intensity and duration of smoking in early adult life. Therefore, these results are only tentative.

With regard to non-alcoholic beverages, we did not observe a significant association with OA. We used a semi-administered FFQ to assess dietary, including non-alcohol beverage, intake during the fourth decade which was assumed to be the important period in the development of OA. Although individuals in this population gave modestly reproducible responses when self-reporting their past beverage intakes, we cannot rule out reporting bias which may explain, in part, the differing associations or lack of association with specific beverages. However, participants were unaware of the hypotheses being tested by the present study so any errors in the classification of the exposure status would be comparable for cases and controls and could lead to underestimation of the reported associations. It is also possible that consumption of alcoholic or non-alcoholic beverages at different periods in adult life may play a role at different stages of the disease (development and progression); therefore, prospective epidemiological studies are required to further investigate these hypotheses.

The mechanisms by which consumption of various beverages may exert their effect in the OA disease process are unclear, however plausible explanations have been proposed. Moderate intake of certain types of alcoholic beverages has been found to confer health benefits. These benefits have been attributed to non-ethanol components in alcoholic beverages such as polyphenols which have been shown to modulate human intestinal microbiota and increase antioxidant activity [23,24]. Recent *in vivo* experiments using human faecal samples have shown regular moderate consumption of red wine polyphenols to inhibit non-beneficial bacteria from human microbiota and significantly increase the growth of select beneficial bacteria such as bifidobacteria [23]. Accumulating evidence also suggests that gut microbiota may play a major role in the pathophysiology of obesity and its related disorders [25]. Evidence from murine knockout models using leptin-deficient mice has shown an increased intestinal mucosal permeability and portal endotoxaemia in genetically obese mice [26]. It is proposed that increased absorption of endotoxins may result as a consequence of compromised intestinal flora and enhanced intestinal mucosal permeability in obese patients [27]. These factors, including high circulating levels of inflammatory cytokines observed in obese patients [27,28], might contribute to the onset and progression of OA. Recent studies also demonstrate the potential chondroprotective action of the polyphenol resveratrol, which is highly present in grape skin and red wine, in preventing cartilage degradation and joint damage in *in vitro* and in animal studies [29-32]. Therefore, the inverse association between wine and OA observed in this study may be partly explained by potential beneficial antioxidant activity and favourable modification of gut microbiota conferred by dietary polyphenols in wine.

How beer might increase the risk of OA remains speculative. There are suggestions that elevated uric acid concentrations may increase the risk of OA [33]. In a recent study [34], serum uric acid levels showed strong correlations with both synovial IL-1 levels and OA severity in patients with knee OA but with no clinical evidence or self-report of gout. Furthermore, increase in serum uric acid levels has been associated with increasing beer intake [35]. We found that increasing beer consumption independently increased the risk of both knee and hip OA.

Furthermore, many people who drink beer often end up with a ‘beer belly’ which may increase the risk of OA via biomechanical loading to the weight bearing joints. However, after adjusting for BMI or the waist-hip-ratio (data not reported), the dose response relationship remained, suggesting that the risk is less likely due to obesity or central obesity but is related to the effects of the high level of beer intake. Beer intake is one of the well-established risk factors for gout because of its high content of purines, especially guanosine [36]. Further evidence shows that people with gout are more likely to have OA or *vice versa* [37]. We did find a higher proportion of people with gout in the OA group (11% in knee OA and 9% in hip OA) than in the control group (7%, *P* <0.004). Gout was obviously a confounding factor for the association. However, after the adjustment for gout and other potential confounding factors, the association and dose response relationship still existed. Nonetheless, we recognise that our results may be affected by unobserved confounders associated with knee and hip OA and with beer consumption. For example, there are suggestions that drinking patterns are correlated with the choice of an alcoholic beverage in different populations and socioeconomic groups [38-40]. In this study population, beer was the most commonly consumed alcoholic drink, with a median intake of 10 half pints of beer per week. However, it is also possible that the typically low intakes of wine (median of three glasses per week) or spirits (median of three tots per week) also suggest that a small sample of this population may have had different drinking patterns (for example, wine intake with meals rather than heavy weekend consumption), and may explain the differing associations with specific alcoholic drinks. Thus, it is probable that our findings may be due to alcohol content rather than components of each type of drink. Further evidence on how specific alcoholic drinks are related to the risk of OA is required.

There are several important limitations to this study. Firstly, it was a hospital based study comprising cases with clinically severe knee or hip OA referred for consideration for surgery and controls who were also referred to hospital, but for IVU examination because of non-musculoskeletal symptoms. Although the characteristics of the controls in this study are comparable with other cohorts, including the Health Survey of England and Norfolk EPIC cohort [41], the generalisability of these findings to community based people with less clinically severe knee or hip OA and to controls who are completely healthy is open to question. Secondly, this was a retrospective case control study. The key exposures of interest, specifically previous alcohol and beverage intake, were self-reported using a questionnaire. This is obviously prone to recall bias and, possibly, attribution bias. Although the second questionnaire survey showed reasonable reliability of the measures, these biases cannot be ruled out. Thirdly, alcohol intakes and other beverage intakes are related to many diseases. We only adjusted for gout because of its well-known positive association with beer [36], and kidney disease because of the high prevalence in the IVU controls. Other comorbidities and their potential confounding in this study have yet to be excluded. Also, although we estimated alcohol intake during adult life which was expected to be before development of OA, a case–control study can only identify associations, and true cause and effect needs to be studied ideally by a prospective cohort study that identifies incident cases.

## Conclusions

Beer consumption was associated with an increased risk of knee and hip OA. Wine consumption, however, was associated with a reduced risk of knee OA. A reduced risk was also seen for spirits in hip OA with a dose response relationship. In contrast, non-alcoholic beverages did not appear to associate with knee and hip OA. Although the mechanisms behind these demonstrated positive and inverse associations with consumption of beer and wine, respectively, remain speculative, further studies appear warranted.

## Additional file

Additional file 1: Table S1. Associations between alcohol consumption and knee OA by gender. **Table S2.** Associations between alcohol consumption and hip OA by gender. **Table S3.** Associations between alcohol consumption and knee OA during adult life. **Table S4.** Associations between alcohol consumption and hip OA during adult life. **Table S5.** Total alcohol intake: full multivariable logistic regression models. **Table S6.** Beer consumption: full multivariable logistic regression models. **Table S7.** Wine consumption: full multivariable logistic regression models. **Table S8.** Spirits consumption: full multivariable logistic regression models.

## Abbreviations

95% CI: 95% confidence Interval
aORs: adjusted odds ratio
BMI: body mass index
EPIC: European Prospective Investigation of Cancer and Nutrition
FFQ: food frequency questionnaire
GOAL study: Genetics of OA and Lifestyle study
IL-1: interleukin-1
IVU: intravenous urography
KL: Kellgren and Lawrence
OA: osteoarthritis
OR: odds ratio
