# Supplementary material for: Beer and wine consumption and risk of knee or hip osteoarthritis: a case control study
Source: Arthritis Res Ther. 2015 Feb 5;17(1):23. doi: 10.1186/s13075-015-0534-4 (PMC4355424; doi:10.1186/s13075-015-0534-4)
Supplement: Additional file 1: Table S1. — Associations between alcohol consumption and knee OA by gender. Table S2. Associations between alcohol consumption and hip OA by gender. Table S3. Associations between alcohol consumption and knee OA during adult life. Table S4. Associations between alcohol consumption and hip OA during adult life. Table S5. Total alcohol intake: full multivariable logistic regression models. Table S6. Beer consumption: full multivariable logistic regression models. Table S7. Wine consumption: full multivariable logistic regression models. Table S8. Spirits consumption: full multivariable logistic regression models. [file 13075_2015_534_MOESM1_ESM.docx]

**SUPPLEMENTARY INFORMATION**

Table 1S: Associations between alcohol consumption and knee OA by gender

| Alcohol consumption/ Frequency of consumption | **Men** | | | | | | | | | | | |  | **Women** | | | | | | | | | | | |
| --- | --- | --- | --- | --- | --- | --- | --- | --- | --- | --- | --- | --- | --- | --- | --- | --- | --- | --- | --- | --- | --- | --- | --- | --- | --- |
|  | Cases | Controls |  | Crude OR | | | |  | Adjusted‡ OR | | | |  | Cases | Controls |  | Crude OR | | | |  | Adjusted‡ OR | | | |
|  | N | N |  | ORs | 95.0% C.I | | P value |  | ORs | 95.0% C.I | | P value |  | N | N |  | ORs | 95.0% C.I | | P value |  | ORs | 95.0% C.I | | P value |
| **Total alcohol intake** |  |  |  |  |  |  |  |  |  |  |  |  |  |  |  |  |  |  |  |  |  |  |  |  |  |
| Nondrinkers | 49 | 59 |  | 1 |  |  |  |  | 1 |  |  |  |  | 231 | 127 |  | 1 |  |  |  |  | 1 |  |  |  |
| <=2per week | 26 | 35 |  | 0.89 | 0.47 | 1.68 | 0.730 |  | 0.82 | 0.39 | 1.74 | 0.603 |  | 76 | 58 |  | 0.72 | 0.48 | 1.08 | 0.112 |  | 0.90 | 0.52 | 1.54 | 0.696 |
| 3-6 per week | 60 | 61 |  | 1.18 | 0.70 | 1.99 | 0.524 |  | 1.00 | 0.54 | 1.85 | 0.989 |  | 75 | 107 |  | **0.39** | **0.27** | **0.56** | **<0.001** |  | 0.69 | 0.42 | 1.16 | 0.162 |
| 7-14 per week | 121 | 113 |  | 1.29 | 0.82 | 2.04 | 0.276 |  | 1.31 | 0.75 | 2.28 | 0.337 |  | 56 | 91 |  | **0.34** | **0.23** | **0.50** | **<0.001** |  | 0.65 | 0.36 | 1.15 | 0.139 |
| >=15 per week | 257 | 212 |  | 1.46 | 0.96 | 2.22 | 0.078 |  | 1.50 | 0.88 | 2.56 | 0.135 |  | 25 | 49 |  | **0.28** | **0.17** | **0.48** | **<0.001** |  | 0.93 | 0.44 | 1.96 | 0.852 |
| P trend |  |  |  |  |  |  | **0.022** |  |  |  |  | **0.029** |  |  |  |  |  |  |  | **<0.001** |  |  |  |  | 0.217 |
| **Beer*** |  |  |  |  |  |  |  |  |  |  |  |  |  |  |  |  |  |  |  |  |  |  |  |  |  |
| None | 62 | 87 |  | 1 |  |  |  |  | 1 |  |  |  |  | 326 | 253 |  | 1 |  |  |  |  | 1 |  |  |  |
| <=3 per week | 45 | 44 |  | 1.44 | 0.85 | 2.43 | 0.180 |  | 1.3 | 0.7 | 2.43 | 0.411 |  | 83 | 86 |  | 0.75 | 0.53 | 1.06 | 0.099 |  | 0.98 | 0.6 | 1.59 | 0.923 |
| 4-7 per week | 68 | 83 |  | 1.15 | 0.73 | 1.82 | 0.550 |  | 1.17 | 0.67 | 2.04 | 0.586 |  | 26 | 56 |  | **0.36** | **0.22** | **0.59** | **<0.001** |  | 0.77 | 0.38 | 1.59 | 0.483 |
| 8 - 19 per week | 162 | 130 |  | **1.75** | **1.17** | **2.61** | **0.006** |  | **2.02** | **1.23** | **3.30** | **0.005** |  | 21 | 32 |  | **0.51** | **0.29** | **0.90** | **0.021** |  | 1.79 | 0.77 | 4.18 | 0.176 |
| >=20 per week | 176 | 136 |  | **1.82** | **1.22** | **2.7** | **0.003** |  | **1.97** | **1.18** | **3.26** | **0.009** |  | 7 | 5 |  | 1.09 | 0.34 | 3.46 | 0.888 |  | 2.70 | 0.64 | 11.3 | 0.176 |
| P trend |  |  |  |  |  |  | **0.001** |  |  |  |  | **0.002** |  |  |  |  |  |  |  | **<0.001** |  |  |  |  | 0.255 |
| **Wine*** |  |  |  |  |  |  |  |  |  |  |  |  |  |  |  |  |  |  |  |  |  |  |  |  |  |
| None | 421 | 343 |  | 1 |  |  |  |  | 1 |  |  |  |  | 349 | 236 |  | 1 |  |  |  |  | 1 |  |  |  |
| <=1 per week | 12 | 14 |  | 0.70 | 0.32 | 1.53 | 0.369 |  | 0.92 | 0.34 | 2.51 | 0.871 |  | 27 | 29 |  | 0.63 | 0.36 | 1.09 | 0.099 |  | 0.57 | 0.27 | 1.21 | 0.143 |
| 2-3 per week | 30 | 36 |  | 0.68 | 0.41 | 1.13 | 0.133 |  | 0.80 | 0.43 | 1.49 | 0.480 |  | 47 | 74 |  | **0.43** | **0.29** | **0.64** | **<0.001** |  | 0.70 | 0.40 | 1.22 | 0.209 |
| 4-6 per week | 26 | 47 |  | **0.45** | **0.27** | **0.74** | **0.002** |  | **0.42** | **0.23** | **0.77** | **0.005** |  | 24 | 56 |  | **0.29** | **0.17** | **0.48** | **<0.001** |  | 0.70 | 0.32 | 1.51 | 0.364 |
| >=7 per week | 24 | 40 |  | **0.49** | **0.29** | **0.83** | **0.008** |  | 0.61 | 0.32 | 1.18 | 0.140 |  | 16 | 37 |  | **0.29** | **0.16** | **0.54** | **<0.001** |  | **0.31** | **0.13** | **0.74** | **0.008** |
| P trend |  |  |  |  |  |  | **<0.001** |  |  |  |  | **0.007** |  |  |  |  |  |  |  | **<0.001** |  |  |  |  | **0.007** |
| **Spirits*** |  |  |  |  |  |  |  |  |  |  |  |  |  |  |  |  |  |  |  |  |  |  |  |  |  |
| None | 402 | 355 |  | 1 |  |  |  |  | 1 |  |  |  |  | 354 | 299 |  | 1 |  |  |  |  | 1 |  |  |  |
| <=1 per week | 26 | 24 |  | 0.96 | 0.54 | 1.70 | 0.880 |  | 1.22 | 0.60 | 2.48 | 0.574 |  | 22 | 19 |  | 0.98 | 0.52 | 1.84 | 0.945 |  | 1.03 | 0.4 | 2.63 | 0.956 |
| 2-3 per week | 16 | 28 |  | **0.50** | **0.27** | **0.95** | **0.034** |  | 0.60 | 0.27 | 1.33 | 0.210 |  | 44 | 48 |  | 0.77 | 0.50 | 1.20 | 0.251 |  | 1.04 | 0.56 | 1.93 | 0.902 |
| 4-7 per week | 28 | 29 |  | 0.85 | 0.50 | 1.46 | 0.562 |  | 0.59 | 0.30 | 1.13 | 0.113 |  | 30 | 35 |  | 0.72 | 0.43 | 1.21 | 0.216 |  | 1.13 | 0.54 | 2.35 | 0.745 |
| >=8 per week | 41 | 44 |  | 0.82 | 0.53 | 1.29 | 0.395 |  | 0.83 | 0.47 | 1.48 | 0.536 |  | 13 | 31 |  | **0.35** | **0.18** | **0.69** | **0.002** |  | 0.80 | 0.31 | 2.07 | 0.648 |
| P trend |  |  |  |  |  |  | 0.153 |  |  |  |  | 0.148 |  |  |  |  |  |  |  | 0.002 |  |  |  |  | 0.964 |

‡Adjusted for age, gender, BMI, smoking, other alcoholic drinks*, energetic physical activities, occupational risks, significant injury, kidney diseases and gout

¶ includes participants who did not drink alcohol on a regular basis (at least 1 alcoholic drink per week) during age period 21-50 years

¥includes alcohol abstainers at ages 21-50 and drinkers of other alcoholic drinks

Table 2S: Associations between alcohol consumption and hip OA by gender

| Alcohol intake / Frequency of consumption | **Men** | | | | | | | | | | | |  | **Women** | | | | | | | | | | | |
| --- | --- | --- | --- | --- | --- | --- | --- | --- | --- | --- | --- | --- | --- | --- | --- | --- | --- | --- | --- | --- | --- | --- | --- | --- | --- |
|  | Cases | Controls |  | Crude OR | | | |  | Adjusted‡ OR | | | |  | Cases | Controls |  | Crude OR | | | |  | Adjusted‡ OR | | | |
|  | N | N |  | ORs | 95.0% C.I | | P value |  | ORs | 95.0% C.I | | P value |  | N | N |  | ORs | 95.0% C.I | | P value |  | ORs | 95.0% C.I | | P value |
| **Total alcohol intake** |  |  |  |  |  |  |  |  |  |  |  |  |  |  |  |  |  |  |  |  |  |  |  |  |  |
| Nondrinkers¶ | 65 | 59 |  | 1 |  |  |  |  | 1 |  |  |  |  | 192 | 127 |  | 1 |  |  |  |  | 1 |  |  |  |
| <=2per week | 17 | 35 |  | **0.44** | **0.22** | **0.87** | **0.018** |  | **0.43** | **0.2** | **0.92** | **0.030** |  | 73 | 58 |  | 0.83 | 0.55 | 1.26 | 0.382 |  | 0.7 | 0.43 | 1.15 | 0.165 |
| 3-6 per week | 54 | 61 |  | 0.80 | 0.48 | 1.34 | 0.399 |  | 0.66 | 0.37 | 1.17 | 0.157 |  | 120 | 107 |  | 0.74 | 0.53 | 1.05 | 0.089 |  | 1.11 | 0.72 | 1.71 | 0.647 |
| 7-14 per week | 99 | 113 |  | 0.80 | 0.51 | 1.24 | 0.312 |  | 0.75 | 0.45 | 1.25 | 0.270 |  | 65 | 91 |  | **0.47** | **0.32** | **0.70** | **<0.001** |  | 0.76 | 0.46 | 1.25 | 0.280 |
| >=15 per week | 251 | 212 |  | 1.07 | 0.72 | 1.60 | 0.722 |  | 1.17 | 0.72 | 1.89 | 0.535 |  | 29 | 49 |  | **0.39** | **0.23** | **0.65** | **<0.001** |  | 0.91 | 0.48 | 1.73 | 0.783 |
| P trend |  |  |  |  |  |  | 0.136 |  |  |  |  | 0.095 |  |  |  |  |  |  |  | **<0.001** |  |  |  |  | 0.663 |
| **Beer*** |  |  |  |  |  |  |  |  |  |  |  |  |  |  |  |  |  |  |  |  |  |  |  |  |  |
| None¥ | 76 | 87 |  | 1 |  |  |  |  | 1 |  |  |  |  | 308 | 253 |  | 1 |  |  |  |  | 1 |  |  |  |
| <=3 per week | 35 | 44 |  | 0.91 | 0.53 | 1.56 | 0.734 |  | 0.8 | 0.44 | 1.47 | 0.471 |  | 84 | 86 |  | 0.8 | 0.57 | 1.13 | 0.209 |  | 0.76 | 0.49 | 1.17 | 0.211 |
| 4-7 per week | 60 | 83 |  | 0.83 | 0.53 | 1.3 | 0.412 |  | 0.92 | 0.55 | 1.54 | 0.759 |  | 61 | 56 |  | 0.89 | 0.60 | 1.33 | 0.585 |  | 1.62 | 0.96 | 2.73 | 0.073 |
| 8 - 19 per week | 131 | 130 |  | 1.15 | 0.78 | 1.71 | 0.475 |  | 1.33 | 0.83 | 2.12 | 0.236 |  | 25 | 32 |  | 0.64 | 0.37 | 1.11 | 0.113 |  | 1.74 | 0.87 | 3.49 | 0.117 |
| >=20 per week | 184 | 136 |  | **1.55** | **1.06** | **2.26** | **0.024** |  | **1.83** | **1.14** | **2.93** | **0.012** |  | 1 | 5 |  | 0.16 | 0.02 | 1.42 | 0.100 |  | 0.15 | 0.01 | 1.58 | 0.114 |
| P trend |  |  |  |  |  |  | **0.006** |  |  |  |  | **0.003** |  |  |  |  |  |  |  | **0.041** |  |  |  |  | 0.257 |
| **Wine*** |  |  |  |  |  |  |  |  |  |  |  |  |  |  |  |  |  |  |  |  |  |  |  |  |  |
| None¥ | 386 | 343 |  | 1 |  |  |  |  | 1 |  |  |  |  | 317 | 236 |  | 1 |  |  |  |  | 1 |  |  |  |
| <=1 per week | 12 | 14 |  | 0.76 | 0.35 | 1.67 | 0.496 |  | 0.95 | 0.38 | 2.36 | 0.910 |  | 25 | 29 |  | 0.64 | 0.37 | 1.12 | 0.121 |  | 0.55 | 0.27 | 1.09 | 0.087 |
| 2-3 per week | 32 | 36 |  | 0.79 | 0.48 | 1.30 | 0.353 |  | 1.01 | 0.56 | 1.82 | 0.979 |  | 70 | 74 |  | 0.70 | 0.49 | 1.02 | 0.062 |  | 0.91 | 0.57 | 1.46 | 0.699 |
| 4-6 per week | 23 | 47 |  | **0.43** | **0.26** | **0.73** | **0.002** |  | **0.45** | **0.25** | **0.83** | **0.011** |  | 38 | 56 |  | **0.51** | **0.32** | **0.79** | **0.003** |  | 0.93 | 0.51 | 1.69 | 0.811 |
| >=7 per week | 33 | 40 |  | 0.73 | 0.45 | 1.19 | 0.208 |  | 0.84 | 0.47 | 1.48 | 0.540 |  | 29 | 37 |  | **0.58** | **0.35** | **0.98** | **0.040** |  | 0.81 | 0.42 | 1.55 | 0.519 |
| P trend |  |  |  |  |  |  | **0.005** |  |  |  |  | 0.097 |  |  |  |  |  |  |  | <0.001 |  |  |  |  | 0.505 |
| **Spirits*** |  |  |  |  |  |  |  |  |  |  |  |  |  |  |  |  |  |  |  |  |  |  |  |  |  |
| None¥ | 392 | 355 |  | 1 |  |  |  |  | 1 |  |  |  |  | 364 | 299 |  | 1 |  |  |  |  | 1 |  |  |  |
| <=1 per week | 14 | 24 |  | 0.53 | 0.27 | 1.04 | 0.064 |  | 0.59 | 0.27 | 1.30 | 0.192 |  | 29 | 19 |  | 1.25 | 0.69 | 2.28 | 0.459 |  | 1.82 | 0.83 | 3.98 | 0.132 |
| 2-3 per week | 24 | 28 |  | 0.78 | 0.44 | 1.36 | 0.379 |  | 0.79 | 0.41 | 1.52 | 0.476 |  | 49 | 48 |  | 0.84 | 0.55 | 1.28 | 0.418 |  | 1.08 | 0.63 | 1.87 | 0.773 |
| 4-7 per week | 30 | 29 |  | 0.94 | 0.55 | 1.59 | 0.809 |  | 0.70 | 0.38 | 1.27 | 0.238 |  | 23 | 35 |  | **0.54** | **0.31** | **0.93** | **0.027** |  | 0.55 | 0.28 | 1.06 | 0.075 |
| >=8 per week | 26 | 44 |  | **0.54** | **0.32** | **0.89** | **0.015** |  | **0.49** | **0.27** | **0.89** | **0.019** |  | 14 | 31 |  | **0.37** | **0.19** | **0.71** | **0.003** |  | 0.65 | 0.28 | 1.47 | 0.297 |
| P trend |  |  |  |  |  |  | **0.021** |  |  |  |  | **0.010** |  |  |  |  |  |  |  | **0.001** |  |  |  |  | 0.157 |

‡Adjusted for age, gender, BMI, smoking, other alcoholic drinks*, energetic physical activities, occupational risks , significant injury, kidney diseases and gout

¶ includes participants who did not drink alcohol on a regular basis (at least 1 alcoholic drink per week) during age period 21-50 years

¥includes alcohol abstainers at ages 21-50 and drinkers of other alcoholic drinks

Table 3S: Associations between alcohol consumption and knee OA during adult life

| Alcohol intake / Frequency of consumption | **Ages 21-30** | | | | | | |  | **Ages 31-40** | | | | | | |  | **Ages 41-50** | | | | | | |
| --- | --- | --- | --- | --- | --- | --- | --- | --- | --- | --- | --- | --- | --- | --- | --- | --- | --- | --- | --- | --- | --- | --- | --- |
|  | Cases | Controls | | Adjusted OR | | | |  | Cases | Controls | | Adjusted OR | | | |  | Cases | Controls | | Adjusted OR | | | |
|  | N | N |  | ORs | 95.0% C.I | | P value |  | N | N |  | ORs | 95.0% C.I | | P value |  | N | N |  | ORs | 95.0% C.I | | P value |
| **Total alcohol intake** |  |  |  |  |  |  |  |  |  |  |  |  |  |  |  |  |  |  |  |  |  |  |  |
| Nondrinkers | 337 | 247 |  | 1 |  |  |  |  | 300 | 209 |  | 1.00 |  |  |  |  | 280 | 186 |  | 1.00 |  |  |  |
| <=2per week | 140 | 113 |  | 0.93 | 0.63 | 1.36 | 0.700 |  | 106 | 93 |  | 0.87 | 0.57 | 1.32 | 0.506 |  | 88 | 101 |  | 0.75 | 0.49 | 1.15 | 0.182 |
| 3-6 per week | 126 | 169 |  | **0.69** | **0.48** | **0.99** | **0.046** |  | 135 | 148 |  | 0.89 | 0.60 | 1.31 | 0.553 |  | 138 | 157 |  | 0.92 | 0.62 | 1.36 | 0.679 |
| 7-14 per week | 115 | 153 |  | 0.80 | 0.53 | 1.20 | 0.286 |  | 139 | 151 |  | 0.88 | 0.59 | 1.32 | 0.542 |  | 162 | 175 |  | 0.91 | 0.62 | 1.34 | 0.640 |
| >=15 per week | 232 | 199 |  | 1.36 | 0.92 | 2.01 | 0.126 |  | 233 | 219 |  | 1.14 | 0.77 | 1.70 | 0.518 |  | 240 | 207 |  | 1.14 | 0.76 | 1.69 | 0.529 |
| P trend | 950 | 881 |  |  |  |  | 0.354 |  | 913 | 820 |  |  |  |  | 0.625 |  | 908 | 826 |  |  |  |  | 0.513 |
| **Beer** |  |  |  |  |  |  |  |  |  |  |  |  |  |  |  |  |  |  |  |  |  |  |  |
| None | 430 | 367 |  | 1.00 |  |  |  |  | 406 | 343 |  | 1.00 |  |  |  |  | 408 | 386 |  | 1.00 |  |  |  |
| <=3 per week | 109 | 105 |  | 0.90 | 0.61 | 1.33 | 0.597 |  | 82 | 83 |  | 0.93 | 0.60 | 1.43 | 0.735 |  | 77 | 92 |  | 1.03 | 0.67 | 1.58 | 0.899 |
| 4-7 per week | 88 | 123 |  | 0.67 | 0.44 | 1.01 | 0.058 |  | 93 | 116 |  | 0.91 | 0.59 | 1.39 | 0.650 |  | 92 | 111 |  | 1.28 | 0.83 | 1.98 | 0.258 |
| 8 - 19 per week | 139 | 130 |  | 1.21 | 0.80 | 1.83 | 0.362 |  | 153 | 135 |  | 1.39 | 0.91 | 2.10 | 0.126 |  | 167 | 115 |  | **2.03** | **1.35** | **3.05** | **0.001** |
| >=20 per week | 184 | 156 |  | 1.49 | 0.99 | 2.26 | 0.058 |  | 179 | 143 |  | **1.79** | **1.16** | **2.76** | **0.008** |  | 164 | 122 |  | **1.97** | **1.28** | **3.02** | **0.002** |
| P trend | 950 | 881 |  |  |  |  | 0.061 |  | 913 | 908 |  |  |  |  | **0.008** |  | 908 | 826 |  |  |  |  | **<0.001** |
| **Wine** |  |  |  |  |  |  |  |  |  |  |  |  |  |  |  |  |  |  |  |  |  |  |  |
| None | 883 | 746 |  | 1.00 |  |  |  |  | 809 | 617 |  | 1.00 |  |  |  |  | 730 | 535 |  | 1.00 |  |  |  |
| <=1 per week | 20 | 30 |  | 0.51 | 0.25 | 1.04 | 0.065 |  | 28 | 27 |  | 1.02 | 0.50 | 2.06 | 0.956 |  | 25 | 35 |  | 0.75 | 0.37 | 1.48 | 0.403 |
| 2-3 per week | 26 | 44 |  | 0.67 | 0.36 | 1.26 | 0.211 |  | 36 | 58 |  | 0.60 | 0.35 | 1.05 | 0.073 |  | 68 | 82 |  | 0.83 | 0.54 | 1.27 | 0.384 |
| 4-6 per week | 15 | 41 |  | 0.58 | 0.26 | 1.27 | 0.172 |  | 25 | 64 |  | **0.45** | **0.24** | **0.84** | **0.012** |  | 47 | 94 |  | **0.56** | **0.35** | **0.91** | **0.019** |
| >=7 per week | 6 | 20 |  | **0.33** | **0.11** | **0.98** | **0.046** |  | 15 | 54 |  | **0.28** | **0.14** | **0.57** | **<0.001** |  | 38 | 80 |  | **0.53** | **0.31** | **0.88** | **0.014** |
| P trend | 950 | 881 |  |  |  |  | **0.004** |  | 913 | 908 |  |  |  |  | **<0.001** |  | 908 | 826 |  |  |  |  | **0.002** |
| **Spirits** |  |  |  |  |  |  |  |  |  |  |  |  |  |  |  |  |  |  |  |  |  |  |  |
| None | 840 | 756 |  | 1.00 |  |  |  |  | 794 | 673 |  | 1.00 |  |  |  |  | 754 | 633 |  | 1.00 |  |  |  |
| <=1 per week | 33 | 25 |  | 0.98 | 0.50 | 1.90 | 0.945 |  | 26 | 26 |  | 0.99 | 0.48 | 2.05 | 0.970 |  | 30 | 31 |  | 0.97 | 0.49 | 1.93 | 0.924 |
| 2-3 per week | 32 | 36 |  | 1.23 | 0.64 | 2.36 | 0.538 |  | 33 | 40 |  | **0.44** | **0.25** | **0.80** | **0.007** |  | 40 | 54 |  | 0.59 | 0.35 | 1.02 | 0.058 |
| 4-7 per week | 24 | 33 |  | 0.61 | 0.32 | 1.17 | 0.138 |  | 30 | 43 |  | 0.61 | 0.33 | 1.12 | 0.112 |  | 43 | 49 |  | 0.66 | 0.39 | 1.14 | 0.135 |
| >=8 per week | 21 | 31 |  | 0.79 | 0.37 | 1.68 | 0.540 |  | 30 | 38 |  | 0.82 | 0.44 | 1.53 | 0.531 |  | 41 | 59 |  | 0.64 | 0.37 | 1.12 | 0.119 |
| P trend | 950 | 881 |  |  |  |  | 0.297 |  | 913 | 820 |  |  |  |  | **0.030** |  | 908 | 826 |  |  |  |  | **0.013** |

‡Adjusted for age, gender, BMI, smoking, other alcoholic drinks*, energetic physical activities, occupational risks , significant injury, kidney diseases and gout

¶ includes participants who did not drink alcohol on a regular basis (at least 1 alcoholic drink per week) during age period 21-50 years

¥includes alcohol abstainers at age period and drinkers of other alcoholic drinks

Table 4S: Associations between alcohol consumption and hip OA during adult life

| Alcohol intake / Frequency of consumption | **Ages 21-30** | | | | | | |  | **Ages 31-40** | | | | | | |  | **Ages 41-50** | | | | | | |
| --- | --- | --- | --- | --- | --- | --- | --- | --- | --- | --- | --- | --- | --- | --- | --- | --- | --- | --- | --- | --- | --- | --- | --- |
|  | Cases | Controls | | Adjusted OR | | | |  | Cases | Controls | | Adjusted OR | | | |  | Cases | Controls | | Adjusted OR | | | |
|  | N | N |  | ORs | 95.0% C.I | | P value |  | N | N |  | ORs | 95.0% C.I | | P value |  | N | N |  | ORs | 95.0% C.I | | P value |
| **Total alcohol intake** |  |  |  |  |  |  |  |  |  |  |  |  |  |  |  |  |  |  |  |  |  |  |  |
| Nondrinkers¶ | 308 | 247 |  | 1 |  |  |  |  | 277 | 209 |  | 1.00 |  |  |  |  | 257 | 186 |  | 1.00 |  |  |  |
| <=2per week | 124 | 113 |  | 0.85 | 0.59 | 1.22 | 0.384 |  | 107 | 93 |  | 0.79 | 0.53 | 1.16 | 0.224 |  | 99 | 101 |  | 0.73 | 0.50 | 1.08 | 0.119 |
| 3-6 per week | 144 | 169 |  | 0.84 | 0.60 | 1.17 | 0.299 |  | 159 | 148 |  | 1.01 | 0.72 | 1.43 | 0.951 |  | 171 | 157 |  | 1.02 | 0.72 | 1.45 | 0.891 |
| 7-14 per week | 131 | 153 |  | 1.01 | 0.70 | 1.46 | 0.957 |  | 136 | 151 |  | 0.89 | 0.61 | 1.28 | 0.519 |  | 140 | 175 |  | 0.79 | 0.55 | 1.13 | 0.201 |
| >=15 per week | 232 | 199 |  | **1.55** | **1.08** | **2.23** | **0.018** |  | 224 | 219 |  | 1.13 | 0.78 | 1.63 | 0.531 |  | 236 | 207 |  | 1.12 | 0.78 | 1.61 | 0.547 |
| P trend | 939 | 881 |  |  |  |  | **0.048** |  | 903 | 820 |  |  |  |  | 0.597 |  | 903 | 826 |  |  |  |  | 0.648 |
| **Beer** |  |  |  |  |  |  |  |  |  |  |  |  |  |  |  |  |  |  |  |  |  |  |  |
| None¥ | 414 | 367 |  | 1.00 |  |  |  |  | 395 | 343 |  | 1.00 |  |  |  |  | 411 | 386 |  | 1.00 |  |  |  |
| <=3 per week | 96 | 105 |  | 0.72 | 0.50 | 1.04 | 0.084 |  | 85 | 83 |  | 0.87 | 0.59 | 1.30 | 0.509 |  | 94 | 92 |  | 1.02 | 0.70 | 1.49 | 0.907 |
| 4-7 per week | 103 | 123 |  | 0.96 | 0.67 | 1.39 | 0.841 |  | 115 | 116 |  | 1.06 | 0.73 | 1.53 | 0.767 |  | 109 | 111 |  | 1.17 | 0.81 | 1.70 | 0.407 |
| 8 - 19 per week | 135 | 130 |  | 1.38 | 0.94 | 2.03 | 0.095 |  | 126 | 135 |  | 1.22 | 0.83 | 1.80 | 0.314 |  | 123 | 115 |  | 1.39 | 0.95 | 2.04 | 0.087 |
| >=20 per week | 191 | 156 |  | **1.85** | **1.26** | **2.72** | **0.002** |  | 182 | 143 |  | **1.88** | **1.26** | **2.79** | **0.002** |  | 166 | 122 |  | **1.81** | **1.22** | **2.66** | **0.003** |
| P trend | 939 | 881 |  |  |  |  | **0.002** |  | 903 | 820 |  |  |  |  | **0.005** |  | 903 | 826 |  |  |  |  | **0.003** |
| **Wine** |  |  |  |  |  |  |  |  |  |  |  |  |  |  |  |  |  |  |  |  |  |  |  |
| None¥ | 836 | 746 |  | 1.00 |  |  |  |  | 752 | 617 |  | 1.00 |  |  |  |  | 672 | 535 |  | 1.00 |  |  |  |
| <=1 per week | 28 | 30 |  | 0.67 | 0.36 | 1.25 | 0.212 |  | 21 | 27 |  | 0.71 | 0.36 | 1.41 | 0.332 |  | 28 | 35 |  | 0.79 | 0.43 | 1.46 | 0.453 |
| 2-3 per week | 44 | 44 |  | 1.05 | 0.62 | 1.77 | 0.862 |  | 61 | 58 |  | 1.01 | 0.64 | 1.59 | 0.962 |  | 79 | 82 |  | 0.93 | 0.63 | 1.36 | 0.701 |
| 4-6 per week | 21 | 41 |  | 0.84 | 0.44 | 1.61 | 0.608 |  | 38 | 64 |  | 0.86 | 0.51 | 1.43 | 0.550 |  | 59 | 94 |  | 0.79 | 0.52 | 1.20 | 0.264 |
| >=7 per week | 10 | 20 |  | 0.45 | 0.19 | 1.07 | 0.069 |  | 31 | 54 |  | 0.64 | 0.38 | 1.09 | 0.101 |  | 65 | 80 |  | 0.88 | 0.58 | 1.33 | 0.548 |
| P trend | 939 | 881 |  |  |  |  | 0.138 |  | 903 | 820 |  |  |  |  | 0.139 |  | 903 | 826 |  |  |  |  | 0.287 |
| **Spirits** |  |  |  |  |  |  |  |  |  |  |  |  |  |  |  |  |  |  |  |  |  |  |  |
| None¥ | 825 | 756 |  | 1.00 |  |  |  |  | 795 | 673 |  | 1.00 |  |  |  |  | 747 | 633 |  | 1.00 |  |  |  |
| <=1 per week | 29 | 25 |  | 0.90 | 0.48 | 1.71 | 0.757 |  | 22 | 26 |  | 0.91 | 0.46 | 1.78 | 0.775 |  | 30 | 31 |  | 0.97 | 0.53 | 1.79 | 0.927 |
| 2-3 per week | 42 | 36 |  | 1.26 | 0.73 | 2.19 | 0.410 |  | 38 | 40 |  | **0.56** | **0.33** | **0.95** | **0.030** |  | 53 | 54 |  | 0.79 | 0.49 | 1.25 | 0.308 |
| 4-7 per week | 23 | 33 |  | 0.57 | 0.31 | 1.06 | 0.076 |  | 32 | 43 |  | **0.57** | **0.33** | **0.98** | **0.041** |  | 38 | 49 |  | **0.59** | **0.36** | **0.96** | **0.035** |
| >=8 per week | 20 | 31 |  | 0.68 | 0.35 | 1.35 | 0.276 |  | 16 | 38 |  | **0.38** | **0.19** | **0.75** | **0.006** |  | 35 | 59 |  | **0.55** | **0.33** | **0.91** | **0.020** |
| P trend | 939 | 881 |  |  |  |  | 0.156 |  | 903 | 820 |  |  |  |  | **<0.001** |  | 903 | 826 |  |  |  |  | **0.002** |

‡Adjusted for age, gender, BMI, smoking, other alcoholic drinks*, energetic physical activities, occupational risks , significant injury, kidney diseases and gout

¶ includes participants who did not drink alcohol on a regular basis (at least 1 alcoholic drink per week) during age period 21-50 years

¥includes alcohol abstainers at age period and drinkers of other alcoholic drinks

Table 5S: Total alcohol intake: Full multivariable logistic regression models

| Variable | Variable definition | **KNEE OA** | | | | | | | | |  | **HIP OA** | | | | | | | | |
| --- | --- | --- | --- | --- | --- | --- | --- | --- | --- | --- | --- | --- | --- | --- | --- | --- | --- | --- | --- | --- |
|  |  | **Model 2** | | | |  | **Model 3** | | | |  | **Model 2** | | | |  | **Model 3** | | | |
|  |  | ORs | 95.0% C.I | | P value |  | ORs | 95.0% C.I | | P value |  | ORs | 95.0% C.I | | P value |  | ORs | 95.0% C.I | | P value |
| Total alcohol intake | Nondrinkers | 1.00 |  |  |  |  | 1.00 |  |  |  |  | 1.00 |  |  |  |  | 1.00 |  |  |  |
|  | <=2per week | 0.90 | 0.61 | 1.31 | 0.571 |  | 0.81 | 0.53 | 1.24 | 0.340 |  | 0.66 | 0.46 | 0.96 | 0.031 |  | 0.63 | 0.42 | 0.94 | 0.025 |
|  | 3-6 per week | 0.76 | 0.54 | 1.07 | 0.111 |  | 0.73 | 0.50 | 1.06 | 0.101 |  | 0.88 | 0.65 | 1.20 | 0.432 |  | 0.88 | 0.63 | 1.24 | 0.468 |
|  | 7-14 per week | 0.95 | 0.68 | 1.32 | 0.745 |  | 0.82 | 0.57 | 1.20 | 0.309 |  | 0.83 | 0.60 | 1.13 | 0.232 |  | 0.75 | 0.53 | 1.06 | 0.106 |
|  | >=15 per week | 1.43 | 1.01 | 2.02 | 0.042 |  | 1.18 | 0.81 | 1.74 | 0.389 |  | 1.42 | 1.03 | 1.96 | 0.030 |  | 1.27 | 0.90 | 1.81 | 0.177 |
| Age | Age | 1.10 | 1.08 | 1.11 | 0.000 |  | 1.09 | 1.08 | 1.11 | 0.000 |  | 1.08 | 1.07 | 1.10 | 0.000 |  | 1.07 | 1.06 | 1.09 | 0.000 |
| Gender | Male vs Female | 1.15 | 0.90 | 1.48 | 0.270 |  | 1.36 | 1.02 | 1.80 | 0.034 |  | 1.56 | 1.24 | 1.97 | 0.000 |  | 1.64 | 1.27 | 2.12 | 0.000 |
| BMI | <25kg/m^2^ | 1.00 |  |  |  |  | 1.00 |  |  |  |  | 1.00 |  |  |  |  | 1.00 |  |  |  |
|  | >25 - <30kg/m^2^ | 3.08 | 2.29 | 4.16 | 0.000 |  | 3.27 | 2.37 | 4.53 | 0.000 |  | 1.59 | 1.24 | 2.03 | 0.000 |  | 1.71 | 1.31 | 2.23 | 0.000 |
|  | ≥30kg/m^2^ | 9.49 | 6.94 | 12.97 | 0.000 |  | 11.25 | 7.95 | 15.93 | 0.000 |  | 3.11 | 2.38 | 4.06 | 0.000 |  | 3.59 | 2.68 | 4.81 | 0.000 |
| Smoking | Never | 1.00 |  |  |  |  | 1.00 |  |  |  |  | 1.00 |  |  |  |  | 1.00 |  |  |  |
|  | Past smoker | 0.61 | 0.48 | 0.78 | 0.000 |  | 0.60 | 0.46 | 0.78 | 0.000 |  | 0.86 | 0.69 | 1.08 | 0.198 |  | 0.86 | 0.67 | 1.10 | 0.230 |
|  | Current smoker | 0.43 | 0.31 | 0.59 | 0.000 |  | 0.50 | 0.34 | 0.72 | 0.000 |  | 0.43 | 0.32 | 0.58 | 0.000 |  | 0.50 | 0.36 | 0.70 | 0.000 |
| Significant joint injury | Yes vs no |  |  |  |  |  | 3.56 | 2.67 | 4.75 | 0.000 |  |  |  |  |  |  | 1.74 | 1.32 | 2.29 | 0.000 |
| Occupational risk factors | No knee OA risk factors |  |  |  |  |  | 1.00 |  |  |  |  |  |  |  |  |  | 1.00 |  |  |  |
|  | 1 knee OA risk factor |  |  |  |  |  | 0.76 | 0.54 | 1.07 | 0.114 |  |  |  |  |  |  | 0.80 | 0.59 | 1.09 | 0.163 |
|  | 2 knee OA risk factors |  |  |  |  |  | 0.90 | 0.65 | 1.25 | 0.537 |  |  |  |  |  |  | 0.76 | 0.56 | 1.03 | 0.077 |
|  | 3 + knee OA risk factors |  |  |  |  |  | 0.90 | 0.65 | 1.23 | 0.490 |  |  |  |  |  |  | 0.73 | 0.54 | 0.98 | 0.037 |
| Kidney disease | Yes vs no |  |  |  |  |  | 0.09 | 0.07 | 0.13 | 0.000 |  |  |  |  |  |  | 0.10 | 0.07 | 0.14 | 0.000 |
| Gout | Yes vs no |  |  |  |  |  | 1.17 | 0.77 | 1.78 | 0.459 |  |  |  |  |  |  | 1.10 | 0.74 | 1.63 | 0.643 |
| Energetic physical activity | Yes vs no |  |  |  |  |  | 1.38 | 1.03 | 1.87 | 0.034 |  |  |  |  |  |  | 1.13 | 0.87 | 1.47 | 0.373 |

Table 6S: Beer consumption: Full multivariable logistic regression models

| Variable | Variable definition | **KNEE OA** | | | | | | | | |  | **HIP OA** | | | | | | | | |
| --- | --- | --- | --- | --- | --- | --- | --- | --- | --- | --- | --- | --- | --- | --- | --- | --- | --- | --- | --- | --- |
|  |  | **Model 2** | | | |  | **Model 3** | | | |  | **Model 2** | | | |  | **Model 3** | | | |
|  |  | ORs | 95.0% C.I | | P value |  | ORs | 95.0% C.I | | P value |  | ORs | 95.0% C.I | | P value |  | ORs | 95.0% C.I | | P value |
| Beer (half pints) | None | 1.00 |  |  |  |  | 1.00 |  |  |  |  | 1 |  |  |  |  | 1.00 |  |  |  |
|  | <=3 per week | 1.09 | 0.78 | 1.51 | 0.617 |  | 1.02 | 0.70 | 1.47 | 0.929 |  | 0.84 | 0.62 | 1.15 | 0.285 |  | 0.79 | 0.56 | 1.11 | 0.169 |
|  | 4-7 per week | 0.92 | 0.64 | 1.33 | 0.674 |  | 0.90 | 0.60 | 1.37 | 0.630 |  | 1.13 | 0.82 | 1.55 | 0.460 |  | 1.15 | 0.81 | 1.64 | 0.435 |
|  | 8 - 19 per week | **1.73** | **1.22** | **2.46** | **0.002** |  | **1.76** | **1.19** | **2.60** | **0.005** |  | **1.44** | **1.03** | **2.00** | **0.030** |  | **1.49** | **1.04** | **2.15** | **0.031** |
|  | >=20 per week | **2.32** | **1.59** | **3.38** | **0.000** |  | **1.93** | **1.26** | **2.94** | **0.002** |  | **2.36** | **1.65** | **3.37** | **0.000** |  | **2.15** | **1.45** | **3.19** | **0.000** |
| Age | Age | 1.10 | 1.08 | 1.12 | 0.000 |  | 1.09 | 1.07 | 1.11 | 0.000 |  | 1.08 | 1.07 | 1.10 | 0.000 |  | 1.07 | 1.06 | 1.09 | 0.000 |
| Gender | Male vs Female | 1.40 | 1.06 | 1.84 | 0.018 |  | 1.77 | 1.29 | 2.44 | 0.000 |  | 1.87 | 1.45 | 2.41 | 0.000 |  | 2.06 | 1.55 | 2.73 | 0.000 |
| BMI | <25kg/m^2^ | 1.00 |  |  |  |  | 1.00 |  |  |  |  | 1.00 |  |  |  |  | 1.00 |  |  |  |
|  | >25 - <30kg/m^2^ | 3.09 | 2.29 | 4.17 | 0.000 |  | 3.33 | 2.40 | 4.62 | 0.000 |  | 1.56 | 1.22 | 2.00 | 0.000 |  | 1.70 | 1.30 | 2.22 | 0.000 |
|  | ≥30kg/m^2^ | 9.41 | 6.88 | 12.88 | 0.000 |  | 11.06 | 7.79 | 15.71 | 0.000 |  | 2.97 | 2.28 | 3.89 | 0.000 |  | 3.48 | 2.59 | 4.67 | 0.000 |
| Smoking | Never | 1.00 |  |  |  |  | 1.00 |  |  |  |  | 1.00 |  |  |  |  | 1.00 |  |  |  |
|  | Past smoker | 0.59 | 0.46 | 0.75 | 0.000 |  | 0.59 | 0.45 | 0.77 | 0.000 |  | 0.82 | 0.66 | 1.03 | 0.084 |  | 0.83 | 0.65 | 1.06 | 0.129 |
|  | Current smoker | 0.40 | 0.29 | 0.56 | 0.000 |  | 0.46 | 0.31 | 0.66 | 0.000 |  | 0.41 | 0.30 | 0.56 | 0.000 |  | 0.47 | 0.34 | 0.66 | 0.000 |
| Significant joint injury | Yes vs no |  |  |  |  |  | 3.65 | 2.73 | 4.89 | 0.000 |  |  |  |  |  |  | 1.79 | 1.35 | 2.36 | 0.000 |
| Occupational risk factors | No knee OA risk factors |  |  |  |  |  | 1.00 |  |  |  |  |  |  |  |  |  | 1.00 |  |  |  |
|  | 1 knee OA risk factor |  |  |  |  |  | 0.76 | 0.54 | 1.07 | 0.115 |  |  |  |  |  |  | 0.82 | 0.60 | 1.12 | 0.204 |
|  | 2 knee OA risk factors |  |  |  |  |  | 0.87 | 0.62 | 1.21 | 0.404 |  |  |  |  |  |  | 0.74 | 0.54 | 1.00 | 0.053 |
|  | 3 + knee OA risk factors |  |  |  |  |  | 0.90 | 0.65 | 1.24 | 0.518 |  |  |  |  |  |  | 0.75 | 0.55 | 1.01 | 0.055 |
| Kidney disease | Yes vs no |  |  |  |  |  | 0.09 | 0.07 | 0.13 | 0.000 |  |  |  |  |  |  | 0.10 | 0.07 | 0.14 | 0.000 |
| Gout | Yes vs no |  |  |  |  |  | 1.43 | 1.06 | 1.94 | 0.020 |  |  |  |  |  |  | 1.16 | 0.89 | 1.51 | 0.282 |
| Energetic physical activity | Yes vs no |  |  |  |  |  | 1.20 | 0.79 | 1.84 | 0.394 |  |  |  |  |  |  | 1.10 | 0.74 | 1.65 | 0.631 |
| Wine (glasses) | None |  |  |  |  |  | 1.00 |  |  |  |  |  |  |  |  |  | 1.00 |  |  |  |
|  | <=1 per week |  |  |  |  |  | 0.72 | 0.40 | 1.28 | 0.259 |  |  |  |  |  |  | 0.67 | 0.39 | 1.14 | 0.140 |
|  | 2-3 per week |  |  |  |  |  | 0.75 | 0.50 | 1.12 | 0.161 |  |  |  |  |  |  | 0.97 | 0.68 | 1.38 | 0.868 |
|  | 4-6 per week |  |  |  |  |  | 0.55 | 0.34 | 0.87 | 0.011 |  |  |  |  |  |  | 0.68 | 0.45 | 1.03 | 0.070 |
|  | >=7 per week |  |  |  |  |  | 0.48 | 0.29 | 0.80 | 0.005 |  |  |  |  |  |  | 0.88 | 0.58 | 1.34 | 0.549 |
| Spirits (tots) | None |  |  |  |  |  | 1.00 |  |  |  |  |  |  |  |  |  | 1.00 |  |  |  |
|  | <=1 per week |  |  |  |  |  | 1.16 | 0.66 | 2.02 | 0.602 |  |  |  |  |  |  | 1.01 | 0.60 | 1.71 | 0.972 |
|  | 2-3 per week |  |  |  |  |  | 0.79 | 0.50 | 1.26 | 0.330 |  |  |  |  |  |  | 0.90 | 0.60 | 1.35 | 0.610 |
|  | 4-7 per week |  |  |  |  |  | 0.77 | 0.48 | 1.23 | 0.272 |  |  |  |  |  |  | 0.63 | 0.41 | 0.98 | 0.040 |
|  | >=8 per week |  |  |  |  |  | 0.80 | 0.49 | 1.30 | 0.370 |  |  |  |  |  |  | 0.53 | 0.33 | 0.85 | 0.008 |

Table 7S Wine consumption: Full multivariable logistic regression models

| Variable | Variable definition | **KNEE OA** | | | | | | | | |  | **HIP OA** | | | | | | | | |
| --- | --- | --- | --- | --- | --- | --- | --- | --- | --- | --- | --- | --- | --- | --- | --- | --- | --- | --- | --- | --- |
|  |  | **Model 2** | | | |  | **Model 3** | | | |  | **Model 2** | | | |  | **Model 3** | | | |
|  |  | ORs | 95.0% C.I | | P value |  | ORs | 95.0% C.I | | P value |  | ORs | 95.0% C.I | | P value |  | ORs | 95.0% C.I | | P value |
| Wine (glasses) | None | 1.00 |  |  |  |  | 1.00 |  |  |  |  | 1.00 |  |  |  |  | 1.00 |  |  |  |
|  | <=1 per week | 0.69 | 0.42 | 1.13 | 0.139 |  | 0.72 | 0.40 | 1.28 | 0.259 |  | 0.68 | 0.42 | 1.09 | 0.111 |  | 0.67 | 0.39 | 1.14 | 0.140 |
|  | 2-3 per week | 0.68 | 0.48 | 0.97 | 0.031 |  | 0.75 | 0.50 | 1.12 | 0.161 |  | 0.83 | 0.60 | 1.14 | 0.242 |  | 0.97 | 0.68 | 1.38 | 0.868 |
|  | 4-6 per week | **0.55** | **0.36** | **0.82** | **0.004** |  | **0.55** | **0.34** | **0.87** | **0.011** |  | **0.63** | **0.44** | **0.91** | **0.013** |  | 0.68 | 0.45 | 1.03 | 0.070 |
|  | >=7 per week | **0.56** | **0.36** | **0.88** | **0.012** |  | **0.48** | **0.29** | **0.80** | **0.005** |  | 0.91 | 0.62 | 1.33 | 0.624 |  | 0.88 | 0.58 | 1.34 | 0.549 |
| Age | Age | 1.08 | 1.07 | 1.10 | 0.000 |  | 1.09 | 1.07 | 1.11 | 0.000 |  | 1.07 | 1.06 | 1.09 | 0.000 |  | 1.07 | 1.06 | 1.09 | 0.000 |
| Gender | Male vs Female | 1.01 | 0.81 | 1.26 | 0.926 |  | 1.77 | 1.29 | 2.44 | 0.000 |  | 1.33 | 1.09 | 1.64 | 0.006 |  | 2.06 | 1.55 | 2.73 | 0.000 |
| BMI | <25kg/m^2^ | 1.00 |  |  |  |  | 1.00 |  |  |  |  | 1.00 |  |  |  |  | 1.00 |  |  |  |
|  | >25 - <30kg/m^2^ | 3.00 | 2.23 | 4.04 | 0.000 |  | 3.33 | 2.40 | 4.62 | 0.000 |  | 1.57 | 1.23 | 2.01 | 0.000 |  | 1.70 | 1.30 | 2.22 | 0.000 |
|  | ≥30kg/m^2^ | 9.39 | 6.87 | 12.84 | 0.000 |  | 11.06 | 7.79 | 15.71 | 0.000 |  | 3.11 | 2.39 | 4.06 | 0.000 |  | 3.48 | 2.59 | 4.67 | 0.000 |
| Smoking | Never | 1.00 |  |  |  |  | 1.00 |  |  |  |  | 1.00 |  |  |  |  | 1.00 |  |  |  |
|  | Past smoker | 0.64 | 0.50 | 0.80 | 0.000 |  | 0.59 | 0.45 | 0.77 | 0.000 |  | 0.88 | 0.71 | 1.10 | 0.269 |  | 0.83 | 0.65 | 1.06 | 0.129 |
|  | Current smoker | 0.43 | 0.31 | 0.59 | 0.000 |  | 0.46 | 0.31 | 0.66 | 0.000 |  | 0.44 | 0.33 | 0.60 | 0.000 |  | 0.47 | 0.34 | 0.66 | 0.000 |
| Significant joint injury | Yes vs no |  |  |  |  |  | 3.65 | 2.73 | 4.89 | 0.000 |  |  |  |  |  |  | 1.79 | 1.35 | 2.36 | 0.000 |
| Occupational risk factors | No knee OA risk factors |  |  |  |  |  | 1.00 |  |  |  |  |  |  |  |  |  | 1.00 |  |  |  |
|  | 1 knee OA risk factor |  |  |  |  |  | 0.76 | 0.54 | 1.07 | 0.115 |  |  |  |  |  |  | 0.82 | 0.60 | 1.12 | 0.204 |
|  | 2 knee OA risk factors |  |  |  |  |  | 0.87 | 0.62 | 1.21 | 0.404 |  |  |  |  |  |  | 0.74 | 0.54 | 1.00 | 0.053 |
|  | 3 + knee OA risk factors |  |  |  |  |  | 0.90 | 0.65 | 1.24 | 0.518 |  |  |  |  |  |  | 0.75 | 0.55 | 1.01 | 0.055 |
| Kidney disease | Yes vs no |  |  |  |  |  | 0.09 | 0.07 | 0.13 | 0.000 |  |  |  |  |  |  | 0.10 | 0.07 | 0.14 | 0.000 |
| Gout | Yes vs no |  |  |  |  |  | 1.43 | 1.06 | 1.94 | 0.020 |  |  |  |  |  |  | 1.16 | 0.89 | 1.51 | 0.282 |
| Energetic physical activity | Yes vs no |  |  |  |  |  | 1.20 | 0.79 | 1.84 | 0.394 |  |  |  |  |  |  | 1.10 | 0.74 | 1.65 | 0.631 |
| Beer (half pints) | None |  |  |  |  |  | 1.00 |  |  |  |  |  |  |  |  |  | 1.00 |  |  |  |
|  | <=3 per week |  |  |  |  |  | 1.02 | 0.70 | 1.47 | 0.929 |  |  |  |  |  |  | 0.79 | 0.56 | 1.11 | 0.169 |
|  | 4-7 per week |  |  |  |  |  | 0.90 | 0.60 | 1.37 | 0.630 |  |  |  |  |  |  | 1.15 | 0.81 | 1.64 | 0.435 |
|  | 8 - 19 per week |  |  |  |  |  | 1.76 | 1.19 | 2.60 | 0.005 |  |  |  |  |  |  | 1.49 | 1.04 | 2.15 | 0.031 |
|  | >=20 per week |  |  |  |  |  | 1.93 | 1.26 | 2.94 | 0.002 |  |  |  |  |  |  | 2.15 | 1.45 | 3.19 | 0.000 |
| Spirits (tots) | None |  |  |  |  |  | 1.00 |  |  |  |  |  |  |  |  |  | 1.00 |  |  |  |
|  | <=1 per week |  |  |  |  |  | 1.16 | 0.66 | 2.02 | 0.602 |  |  |  |  |  |  | 1.01 | 0.60 | 1.71 | 0.972 |
|  | 2-3 per week |  |  |  |  |  | 0.79 | 0.50 | 1.26 | 0.330 |  |  |  |  |  |  | 0.90 | 0.60 | 1.35 | 0.610 |
|  | 4-7 per week |  |  |  |  |  | 0.77 | 0.48 | 1.23 | 0.272 |  |  |  |  |  |  | 0.63 | 0.41 | 0.98 | 0.040 |
|  | >=8 per week |  |  |  |  |  | 0.80 | 0.49 | 1.30 | 0.370 |  |  |  |  |  |  | 0.53 | 0.33 | 0.85 | 0.008 |

Table 8S Spirits consumption: Full multivariable logistic regression models

| Variable | Variable definition | **KNEE OA** | | | | | | | | |  | **HIP OA** | | | | | | | | |
| --- | --- | --- | --- | --- | --- | --- | --- | --- | --- | --- | --- | --- | --- | --- | --- | --- | --- | --- | --- | --- |
|  |  | **Model 2** | | | |  | **Model 3** | | | |  | **Model 2** | | | |  | **Model 3** | | | |
|  |  | ORs | 95.0% C.I | | P value |  | ORs | 95.0% C.I | | P value |  | ORs | 95.0% C.I | | P value |  | ORs | 95.0% C.I | | P value |
| Spirits (tots) | None | 1.00 |  |  |  |  | 1.00 |  |  |  |  | 1.00 |  |  |  |  | 1.00 |  |  |  |
|  | <=1 per week | 1.03 | 0.64 | 1.67 | 0.895 |  | 1.16 | 0.66 | 2.02 | 0.602 |  | 0.86 | 0.54 | 1.38 | 0.534 |  | 1.01 | 0.60 | 1.71 | 0.972 |
|  | 2-3 per week | 0.76 | 0.51 | 1.14 | 0.189 |  | 0.79 | 0.50 | 1.26 | 0.330 |  | 0.91 | 0.63 | 1.31 | 0.612 |  | 0.90 | 0.60 | 1.35 | 0.610 |
|  | 4-7 per week | 0.99 | 0.65 | 1.50 | 0.954 |  | 0.77 | 0.48 | 1.23 | 0.272 |  | 0.84 | 0.56 | 1.25 | 0.391 |  | **0.63** | **0.41** | **0.98** | **0.040** |
|  | >=8 per week | 0.81 | 0.53 | 1.23 | 0.325 |  | 0.80 | 0.49 | 1.30 | 0.370 |  | **0.59** | **0.39** | **0.91** | **0.017** |  | **0.53** | **0.33** | **0.85** | **0.008** |
| Age | Age | 1.09 | 1.07 | 1.11 | 0.000 |  | 1.09 | 1.07 | 1.11 | 0.000 |  | 1.07 | 1.06 | 1.09 | 0.000 |  | 1.07 | 1.06 | 1.09 | 0.000 |
| Gender | Male vs Female | 0.98 | 0.79 | 1.22 | 0.874 |  | 1.77 | 1.29 | 2.44 | 0.000 |  | 1.28 | 1.05 | 1.57 | 0.016 |  | 2.06 | 1.55 | 2.73 | 0.000 |
| BMI | <25kg/m^2^ | 1.00 |  |  |  |  | 1.00 |  |  |  |  | 1.00 |  |  |  |  | 1.00 |  |  |  |
|  | >25 - <30kg/m^2^ | 3.04 | 2.26 | 4.10 | 0.000 |  | 3.33 | 2.40 | 4.62 | 0.000 |  | 1.60 | 1.25 | 2.04 | 0.000 |  | 1.70 | 1.30 | 2.22 | 0.000 |
|  | ≥30kg/m^2^ | 9.74 | 7.13 | 13.30 | 0.000 |  | 11.06 | 7.79 | 15.71 | 0.000 |  | 3.19 | 2.45 | 4.16 | 0.000 |  | 3.48 | 2.59 | 4.67 | 0.000 |
| Smoking | Never | 1.00 |  |  |  |  | 1.00 |  |  |  |  | 1.00 |  |  |  |  | 1.00 |  |  |  |
|  | Past smoker | 0.64 | 0.51 | 0.81 | 0.000 |  | 0.59 | 0.45 | 0.77 | 0.000 |  | 0.91 | 0.73 | 1.14 | 0.420 |  | 0.83 | 0.65 | 1.06 | 0.129 |
|  | Current smoker | 0.46 | 0.33 | 0.63 | 0.000 |  | 0.46 | 0.31 | 0.66 | 0.000 |  | 0.47 | 0.35 | 0.64 | 0.000 |  | 0.47 | 0.34 | 0.66 | 0.000 |
| Significant joint injury | Yes vs no |  |  |  |  |  | 3.65 | 2.73 | 4.89 | 0.000 |  |  |  |  |  |  | 1.79 | 1.35 | 2.36 | 0.000 |
| Occupational risk factors | No knee OA risk factors |  |  |  |  |  | 1.00 |  |  |  |  |  |  |  |  |  | 1.00 |  |  |  |
|  | 1 knee OA risk factor |  |  |  |  |  | 0.76 | 0.54 | 1.07 | 0.115 |  |  |  |  |  |  | 0.82 | 0.60 | 1.12 | 0.204 |
|  | 2 knee OA risk factors |  |  |  |  |  | 0.87 | 0.62 | 1.21 | 0.404 |  |  |  |  |  |  | 0.74 | 0.54 | 1.00 | 0.053 |
|  | 3 + knee OA risk factors |  |  |  |  |  | 0.90 | 0.65 | 1.24 | 0.518 |  |  |  |  |  |  | 0.75 | 0.55 | 1.01 | 0.055 |
| Kidney disease | Yes vs no |  |  |  |  |  | 0.09 | 0.07 | 0.13 | 0.000 |  |  |  |  |  |  | 0.10 | 0.07 | 0.14 | 0.000 |
| Gout | Yes vs no |  |  |  |  |  | 1.43 | 1.06 | 1.94 | 0.020 |  |  |  |  |  |  | 1.16 | 0.89 | 1.51 | 0.282 |
| Energetic physical activity | Yes vs no |  |  |  |  |  | 1.20 | 0.79 | 1.84 | 0.394 |  |  |  |  |  |  | 1.10 | 0.74 | 1.65 | 0.631 |
| Beer (half pints) | None |  |  |  |  |  | 1.00 |  |  |  |  |  |  |  |  |  | 1.00 |  |  |  |
|  | <=3 per week |  |  |  |  |  | 1.02 | 0.70 | 1.47 | 0.929 |  |  |  |  |  |  | 0.79 | 0.56 | 1.11 | 0.169 |
|  | 4-7 per week |  |  |  |  |  | 0.90 | 0.60 | 1.37 | 0.630 |  |  |  |  |  |  | 1.15 | 0.81 | 1.64 | 0.435 |
|  | 8 - 19 per week |  |  |  |  |  | 1.76 | 1.19 | 2.60 | 0.005 |  |  |  |  |  |  | 1.49 | 1.04 | 2.15 | 0.031 |
|  | >=20 per week |  |  |  |  |  | 1.93 | 1.26 | 2.94 | 0.002 |  |  |  |  |  |  | 2.15 | 1.45 | 3.19 | 0.000 |
| Wine (glasses) | None |  |  |  |  |  | 1.00 |  |  |  |  |  |  |  |  |  | 1.00 |  |  |  |
|  | <=1 per week |  |  |  |  |  | 0.72 | 0.40 | 1.28 | 0.259 |  |  |  |  |  |  | 0.67 | 0.39 | 1.14 | 0.140 |
|  | 2-3 per week |  |  |  |  |  | 0.75 | 0.50 | 1.12 | 0.161 |  |  |  |  |  |  | 0.97 | 0.68 | 1.38 | 0.868 |
|  | 4-6 per week |  |  |  |  |  | 0.55 | 0.34 | 0.87 | 0.011 |  |  |  |  |  |  | 0.68 | 0.45 | 1.03 | 0.070 |
|  | >=7 per week |  |  |  |  |  | 0.48 | 0.29 | 0.80 | 0.005 |  |  |  |  |  |  | 0.88 | 0.58 | 1.34 | 0.549 |
